# An enhanced three-stage model for sodium storage in hard carbons†

**DOI:** 10.1039/d4ee06029f

**Published:** 2025-06-05

**Authors:** Enis Oğuzhan Eren, Evgeny Senokos, Ernesto Scoppola, Zihan Song, Markus Antonietti, Paolo Giusto

**Affiliations:** a Department of Colloid Chemistry, Max Planck Institute of Colloids and Interfaces Potsdam 14476 Germany enis.eren@mpikg.mpg.de paolo.giusto@mpikg.mpg.de; b Department of Biomaterials, Max Planck Institute of Colloids and Interfaces Potsdam 14476 Germany; c Department of Engineering Science, University of Oxford Oxford OX1 3PJ UK

## Abstract

A comprehensive understanding of the sodium storage mechanism in hard carbons is essential for developing more efficient anode materials and improving the electrochemical performance of sodium-ion batteries. The mechanism has been the subject of ongoing debate, particularly regarding the role of intercalation, which we found to be insignificant in our study. By combining electrochemical analyses with *operando* characterization techniques, we propose a refined model of sodium storage in hard carbons. Our findings reveal a three-stage process: first, a fast-capacitive mechanism dominates in the slope region; second, a transition phase occurs at the early plateau, where faradaic processes become significant at the carbon micropore inner surface; and finally, micro- and slit-pore filling becomes dominant at the late plateau, driven by a multilayer-like deposition of quasimetallic sodium in the micropores. We believe this refined mechanism promotes a better understanding of the sodium storage mechanism in hard carbons and provides the basis for the rational design of carbon anode materials with superior performance for sodium-ion batteries.

## Abstract 2

A multi-technique *operando* study reveals a three-stage sodium storage mechanism in hard carbon—surface adsorption, accumulation, and pore filling—while classical intercalation is found to be insignificant under practical conditions.

## Graphical abstract

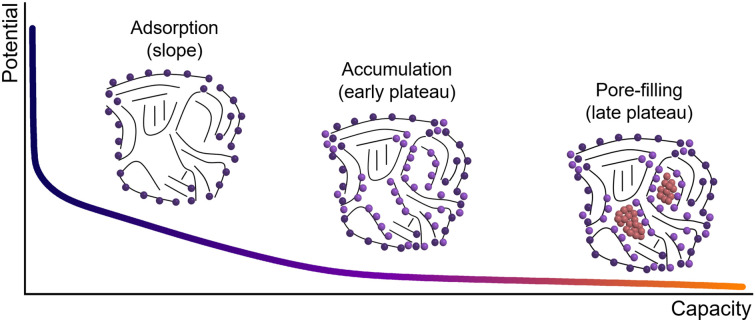

**Broader context**

The global transition to renewable energy and electric transportation depends on the development of efficient, sustainable, and cost-effective energy storage solutions. Sodium-ion batteries are emerging as a promising alternative to lithium-ion batteries, with hard carbons gaining attention as anode materials due to their unique structural properties. These properties facilitate sodium storage through mechanisms that differ from lithium intercalation in graphite.

By combining electrochemical analyses with *operando* small-angle X-ray scattering, wide-angle X-ray scattering, and Raman spectroscopy, we reveal a refined three-stage adsorption-accumulation-filling model: (1) a fast-capacitive mechanism dominates in the slope region, (2) a transition phase occurs at the early plateau, where faradaic processes become significant at the carbon micropore inner surface, resulting in quasimetallic sodium monolayer formation, and (3) micro- and slit-pore filling becomes dominant at the late plateau, driven by a multilayer-like clustering of quasimetallic sodium in the micropores. Additionally, we demonstrate that sodium intercalation is unlikely to play a critical role in the overall sodium storage mechanism. By addressing challenges in understanding the electrochemical evolution, this work contributes to the development of more sustainable energy storage technologies and advances battery research.

## 1. Introduction

The sodium storage mechanism in hard carbons (HCs) has long been studied due to their potential for commercial-scale sodium-ion batteries (SIBs).^1–3^ These materials feature a localized short-range arrangement, often referred to as a pseudo-graphitic structure, that is rich in defect sites, and forms a large fraction of isolated closed pores.^4–6^ The sodium storage mechanism in hard carbon differs significantly from the lithium storage mechanism in graphite, necessitating unique material properties for being effective in SIBs. The similarities drawn from lithium-ion battery (LIB) chemistry do not directly apply to SIBs due to the absence of a stable sodium–carbon intercalation compound.^7^ Theoretical calculations propose a stable sodium–carbon compound NaC_64_ (35 mA h g^−1^), which represents an insignificant capacity compared to LiC_6_ (372 mA h g^−1^), and therefore, challenges the feasibility of intercalation-based sodium storage mechanism in such materials.^8^ Consequently, the emphasis is shifting towards pore engineering,^9^ particularly by controlling the ratio of open to closed pores to enhance plateau capacity and overall sodium storage performance,^10–12^ making it essential to understand the underlying sodium storage mechanisms in order to enable SIBs to compete with state-of-the-art LIBs.

The widely accepted sodium storage mechanism in hard carbon materials is centered around the adsorption-intercalation/pore-filling model, which is typically divided into two main potential regions in the galvanostatic charge/discharge (GCD) profile.^13–17^ The first, known as the “sloping region,” occurs at the onset of the GCD curve and is associated with capacitive storage caused by ion adsorption at favorable sites such as surfaces, edges, and structural defects.^13^ The second, the diffusion-limited “plateau region,” exhibits a nearly constant voltage profile close to 0 V (*vs.* Na^+^/Na) and accounts for bulk sodium storage. However, a detailed understanding of the storage mechanism remains elusive, and multiple competing or complementary models have been proposed. These include insertion–adsorption,^18^ adsorption-insertion,^19^ adsorption-nanopore filling,^20^ and combined adsorption-nanopore filling-intercalation models.^21,22^ The ambiguity primarily lies in the interpretation of the plateau region, which remains highly material-specific and is influenced by factors such as pore size distribution, defect density, electronic properties, and thermal treatment.^22,23^ This complexity highlights the need for further detailed studies to optimize hard carbon design for practical SIB applications.

Such complex phenomena can qualitatively be elucidated through complementary *operando*/*in situ* characterization techniques, which provide insight into the actual alterations and reactions occurring within the anode material during electrochemical operation.^24^ Synchrotron X-ray scattering methods, in particular, offer high-throughput data and time resolution, enabling *operando* experiments for a broad overview of changes happening in the material.^25^ Small-angle X-ray scattering (SAXS) is a method for investigating variation in electron density difference in hard carbons, providing information from bulk porosity to interfacial changes.^21,26^ This method has previously been used to investigate structural changes in battery materials, including early *in situ* studies and more recent *ex situ* analyses of sodium-ion systems.^27,28^ However, these approaches have been limited by either long acquisition times or the complexities of sample preparation, making it difficult to capture real-time dynamics. The introduction of synchrotron-based *operando* SAXS enables direct observation of the electrochemical processes during cycling.^21,26^ This advancement allows for high-resolution tracking of pore-level changes in hard carbon anodes under true operating conditions and enables monitoring across multiple electrode locations, providing a more representative view of the sodium storage mechanism.

Additionally, wide-angle X-ray scattering (WAXS) allows for monitoring changes happening at the lattice level. For example, the strain induced by alkali ion insertion can be monitored if the stacking distance of the local arrangement changes. On the other hand, Raman spectroscopy can provide insights into changes in carbon bond vibrations, potentially deconvoluting the effects of electrochemical processes.^29^

**Fig. 1:**
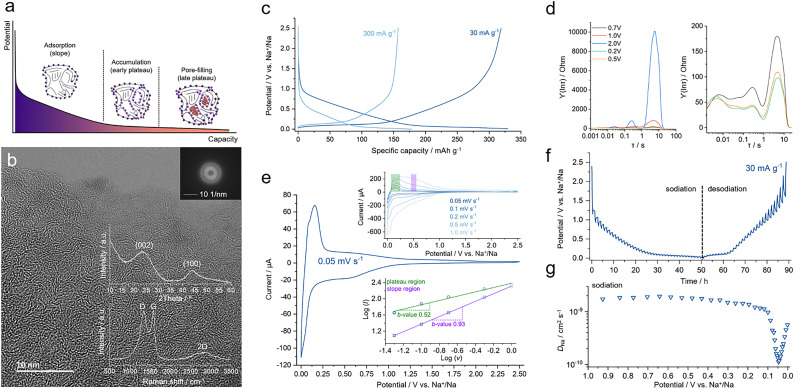
(a) Schematic representation of the proposed three-stage sodium storage mechanism in HCs. (b) HRTEM image of the HC, showing turbostratic domains. Inset: Corresponding FFT pattern and structural characterization *via* XRD and Raman spectroscopy. (c) GCD profiles at low (30 mA g^−1^) and high (300 mA g^−1^) current densities, demonstrating typical sloping and plateau behavior. (d) DRT analysis at different potentials, revealing interfacial resistance and kinetic evolution across the slope region. (e) CV curves at various scan rates and corresponding *b*-value analysis for slope and plateau regions. (f) GITT profile of the HC during sodiation and desodiation at 30 mA g^−1^. (g) Sodium-ion diffusion coefficients derived from the GITT data.

Based on these, we propose an updated and more holistic three-stage model for the sodium storage mechanism in microporous hard carbons, systematically combining electrochemical characterizations with *operando* SAXS, WAXS, and Raman spectroscopy. Initially, the surface-driven capacitive mechanism for sodium ion adsorption dominates in the slope region. We propose that the plateau region encompasses a two-stage mechanism, divided into early and late plateau stages (Fig. 1a). During the early plateau, as sodium ions accumulate at the available sites, faradaic charge transfer reactions begin. However, the repulsion effect between the sodium ions slows the kinetics during this stage.^30^ In the late plateau, as sodium ions become less positively charged, the repulsion effect is reduced, allowing quasimetallic sodium clustering to occur in the micropores. This facilitates the micropore-filling mechanism and leads to a sharp increase in diffusion coefficients. We anticipate that the adsorption-accumulation-filling model for this class of materials reflects a smooth transition in each region instead of sharp changes usually observed in purely insertion mechanisms. The inhomogeneous nature of hard carbons poses challenges in achieving the desired structural and physico-chemical properties for efficient sodium storage. We believe the results presented here advocate and provide critical insights for the development of more efficient carbonaceous electrode materials for the next generation of SIBs.

## 2. Results and discussion

### 2.1. Physicochemical and electrochemical features of the material

To explore the mechanism of sodium storage, we used a hard carbon material (abbreviated as HC) previously reported by our group, obtained from the thermal condensation of the oligo-EDOT precursor at 1000 °C.^26^ At the nanoscale, HC displays pseudo-graphitic domains with a short-range order, as confirmed by high-resolution transmission electron microscopy (HRTEM) (Fig. 1b). The fast Fourier transform (FFT) shows broad diffuse rings, indicating the absence of long-range order at this scale (Fig. 1b; inset). However, sequential features resembling pseudo-graphitic domains appear, which are characteristic of turbostratic and ramen-like structures.^31^ X-ray diffraction (XRD) patterns (Fig. 1b; inset) exhibit two broad peaks corresponding to the (002) and (100) features of hard carbon materials.^32,33^ By applying Bragg's law to the highest intensity of the (002) peak, an average interlayer distance of 0.375 (±0.01) nm is determined. The Raman spectrum (Fig. 1b; inset) displays typical first-order G (1580 cm^−1^) and D (1345 cm^−1^) bands, representing in-plane phonons with E_2g_ and disorder-oriented A_1g_ symmetries.^34^ Additionally, we observe a broad 2D region typically occurring for pseudo-graphitic structures.^35^ The peak intensity ratio for the D- and G-bands (*I*_D_/*I*_G_) is calculated to be around 1.0, in good agreement with previously reported values for short-range ordered carbonaceous materials.^4,34,36,37^

The sodium storage performance is evaluated through GCD measurements in a half-cell configuration, revealing two distinct regions (Fig. 1c). The first region, commonly referred to as the slope region, occurs at higher potentials (*vs.* Na^+^/Na) and is widely attributed in the literature to a capacitive mechanism, dominated by sodium adsorption at favorable sites such as surfaces, edges, and structural defects.^13,15,16^ The slope region in hard carbon can be further differentiated into two electrochemically distinct sub-stages, as also suggested by previous studies.^13,38^ In the high-potential range (*ca.* 2.5–0.8 V (*vs.* Na^+^/Na)), the electrochemical behavior is primarily governed by interface-driven processes. During this stage, sodium storage involves the rearrangement of ions and counterions at the electrode–electrolyte interface and occupation of weakly bound surface adsorption sites. During this stage, sodium ions begin to migrate through the pre-formed SEI layer, but the system exhibits relatively high impedance due to interfacial resistance and transport limitations.^13,38^

To resolve the overlapping kinetic contributions in this region, we employed distribution of relaxation times (DRT) analysis, which deconvolutes electrochemical impedance spectra into discrete relaxation processes characterized by specific time constants.^39^ As shown in Fig. 1d, the DRT profile at 2.0 V (*vs.* Na^+^/Na) displays a dominant relaxation peak centered at around ∼10 s, indicating significant mass transport resistance and sluggish interfacial kinetics.^38^ As the sodiation potential decreases (*vs*. Na^+^/Na), the peak shifts toward shorter relaxation times and its intensity diminishes, reflecting a transition to faster interfacial dynamics. In the lower slope region (*ca.* 0.8–0.12 V *vs.* Na^+^/Na), sodium ions begin to interact more strongly with the carbon scaffold, occupying higher-energy adsorption sites such as edge planes, structural defects, and near-pore environments. The DRT profiles in this regime become nearly featureless (Fig. 1d), indicating a substantial reduction in impedance and the stabilization of ionic transport pathways as the system approaches the plateau region.^40^

The second prominent region is a diffusion-limited plateau that appears at lower potentials (*vs.* Na^+^/Na) (Fig. 1c). The transition point between the slope and plateau regions was defined at 0.12 V (*vs.* Na^+^/Na), based on the inflection observed in the GCD curve and the derivative capacity (d*Q*/d*V*) plot (Fig. S1a, ESI†). At a current density of 30 mA g^−1^, HC provides a reversible specific capacity of around 320 mA h g^−1^, with a plateau capacity of around 150 mA h g^−1^. A pronounced decrease of capacity at higher current densities (300 mA g^−1^) indicates that the rate performance is limited by diffusional processes occurring in the anode. The sodium storage kinetics is further investigated through cyclic voltammetry (CV) analysis at various scan rates (Fig. 1e). In cases where the redox reaction of the electroactive species mostly depends on diffusion, the concentration of sodium ions at the electrode–electrolyte interface typically varies with time.^41^ To further understand the sodium storage kinetics, we analyzed the relationship between the current and scan rate using CV at various scan rates. The slope of the log(*i*) *versus* log(*v*) plot (commonly referred to as the *b*-value) provides insight into the dominant charge storage mechanism: a *b*-value close to 1 indicates surface-controlled (capacitive) processes, while a *b*-value near 0.5 suggests diffusion-limited behavior typical of bulk storage.^41^ In the plateau region, where the redox peaks are located close to 0 V (*vs.* Na^+^/Na), the *b*-value is 0.52, indicating semi-infinite diffusion kinetics of bulk storage. In contrast, at a fixed potential within the slope region (*e.g.*, 0.5 V *vs.* Na^+^/Na), the calculated *b*-value approaches 0.93 (Fig. 1e; inset). While we note that the Randles–Ševčík equation is typically applied to peak current analysis and is not strictly valid in purely capacitive regions, the near-unity *b*-value nevertheless serves as empirical evidence of surface-controlled kinetics. These findings confirm that the slope region is primarily governed by fast surface-driven processes, while the plateau region is controlled by slower diffusion-limited sodium storage mechanisms.

The sodium-ion effective diffusion coefficients (*D*_Na_) were determined using the galvanostatic intermittent titration technique (GITT) (Fig. 1f), as described in Supplementary Note 1 (ESI†). During both sodiation and desodiation processes, the diffusion coefficients of sodium ions in the electrodes are in the range of 10^−8^ to 10^−9^ cm^2^ s^−1^, consistent with values previously reported for hard carbons (Fig. 1g and Fig. S1d, ESI†).^42–44^ A sharp decrease in diffusion coefficients starts at the transition between slope and plateau regions. Noticeably, *D*_Na_ exhibits a peak in the middle of the plateau region, reaching a minimum value at around 0.05 V (*vs.* Na^+^/Na) and rapidly rising at lower potentials. This behavior points to the existence of an additional transition stage in the sodium storage mechanism, dividing the entire plateau region into two distinct phases: an early plateau and a late plateau. At the onset of the early plateau, following the capacitive adsorption process, sodium ions accumulate at available sites across the carbon surface. As these regions become saturated, the increasing local concentration of sodium ions gives rise to significant electrostatic repulsion, which can reduce ion mobility. This behavior is consistent with previously reported mechanisms describing the repulsion-induced decrease in the diffusion coefficient during the early stages of the plateau.^30^ Furthermore, high sodium ion concentrations decrease the gradient in chemical potential, reducing the driving force for further ion migration into the pore and further slowing diffusion. To better understand the correlation between these results and pore-filling, we conducted *operando* SAXS measurements.

### 2.2. Tracking the pore-filling mechanism with *operando* SAXS

SAXS is a useful method for investigating the structural features of materials within nano- to macro-scale.^45,46^ In our previous study, we introduced a method to conduct *operando* SAXS for carbonaceous electrode materials in sodium-ion batteries, validating the pore-filling sodium storage mechanism in our material.^26^ Here, a custom half-cell is used to conduct these measurements with a synchrotron light source, providing much higher time resolution and sensitivity compared to lab-scale X-ray sources.^26^ The scattering signal can be expressed by the scattering length density (SLD) difference (Δ*ρ*) between objects and their surrounding media.^26,46^ Further experimental information, equations outlining the fitting model, and reproducibility data can be found in Supplementary Note 2 (ESI†).

**Fig. 2:**
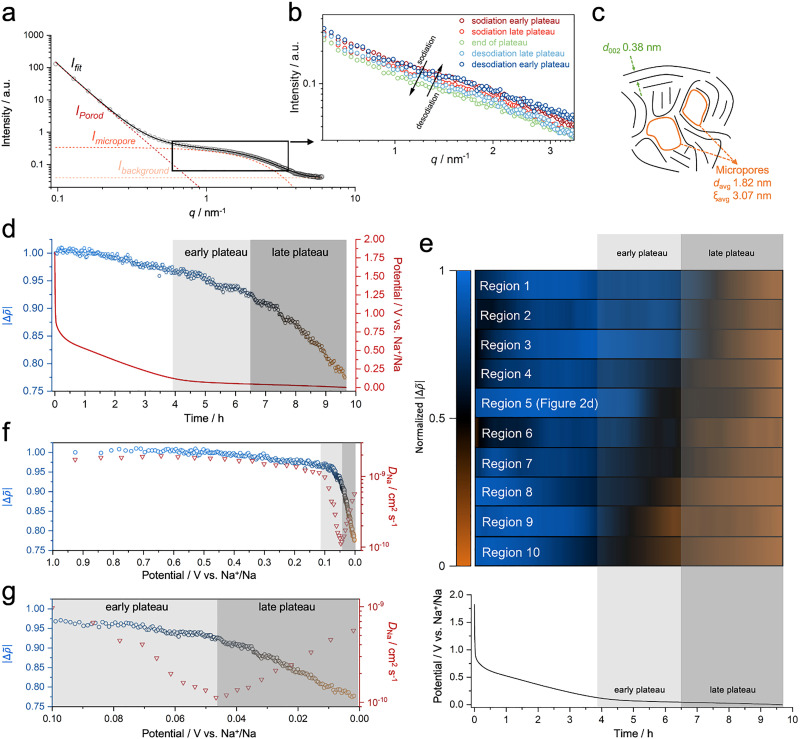
(a) SAXS profile of the HC showing Porod's and the microporous region, along with the fitting of a high-confidence model introduced for non-graphitic carbons. (b) Intensity profile of the mid-*q* region during sodiation and desodiation processes, describing the intensity change that vastly occurs during the late plateau phase. (c) Schematic describing the average interlayer distance, pore diameter, and correlation length. (d) Variation in Δ*ρ̃* during sodiation, revealing pore-filling mostly pronounced at the late plateau region. (e) A heat map visualizes ten different regions in the same electrode for the reproducibility of the analysis. Regions have a similar trend in normalized Δ*ρ̃ (*smoothed) variation. (f) Plot of Δ*ρ̃* with the diffusion coefficients obtained from GITT analysis *versus* the potential. (g) A close-up view of the trends in Δ*ρ̃* and the diffusion coefficient at the early and late plateau.

In general, the SAXS pattern of the HC electrode displays two main regions (Fig. 2a). The first region at low *q* values, called Porod's region, is linked to scattering from interfaces of macroscopic surfaces, exhibiting a distinct slope close to *q*^−4^ at small angles. This is followed by a region at intermediate *q* ranges (*i.e.*, 0.5 to 3 nm^−1^), often associated with the broad distribution of micropores.^46^ Upon sodiation, a noticeable decrease in intensity is observed within this mid-*q* region (Fig. 2b). This decrease becomes pronounced in the late plateau, which can be interpreted as a signature of sodium ion diffusion into the micropores. Initially, the micropores are empty with a relatively low electron density. As sodium ions enter these pores, they increase the electron density within the confined spaces. This reduces the contrast in electron density between the pore walls and the pore volume, causing a decrease in SAXS intensity. Upon desodiation, the intensity returns to its initial level, indicating the reversibility of the pore-filling mechanism. This initial observation is further elucidated by applying a high-confidence fitting model to the data.^26^ Additionally, the electrode is probed in ten different spots to provide statistical information. This approach is necessary due to the inhomogeneous nature of the hard carbon, which requires more input to establish solid quantification. In these regions, the average correlation length (*ξ*) and average pore diameter (*d*_a_) values are calculated as 3.07 and 1.82 nm, respectively (Fig. 2c).

The unitless quantity of relative electron density variation Δ*ρ̃* can be extracted from the fitting model.^26^ Here, it denotes the normalized ratio of two distinct electron density differences assigned to micropores and the carbon matrix. A detailed explanation of this quantity can be found in Supplementary Note 2 (ESI†). The slope region exhibits little to negligible effect on Δ*ρ̃*, suggesting that the capacitive sodium storage does not significantly impact the changes occurring in micropores, as expected. When sodium ions diffuse into the micropores, Δ*ρ̃* is expected to decrease as the electrons carried by the sodium ions create an additional contrast that decreases the electron density variation arising from micropores. This can be evaluated as direct evidence of a pore-filling sodium storage mechanism.^26^

During the early plateau region, Δ*ρ̃* remains stable or shows a slight decrease, which can be partly attributed to the formation of the monolayer at the pore surfaces and entrances, where faradaic charge transfer begins (Fig. 2d). At this stage, quasimetallic clustering in pore volume is low or negligible. Upon reaching the late plateau, Δ*ρ̃* decreases sharply, indicating the dominance of the pore-filling mechanism during this stage (Fig. 2d and Fig. S3, ESI†). This phenomenon is consistent across the ten different areas of the electrode (Fig. 2e), where a sharp decrease in the Δ*ρ̃* mainly occurs during the late plateau. However, in some areas of the electrode, we noticed the pore-filling starts slightly earlier, and we attribute this to minor differences occurring between different areas of the casted electrode (*i.e.*, Regions 8, 9, and 10).

Despite the average pore diameters being relatively similar across the ten areas studied, as estimated from the SAXS model, there is a significant variation in the correlation length between each measurement spot (Table S1, ESI†). However, within these ten areas (Fig. 2e), no relationship is observed between the correlation length and the pore-filling dynamics. Additionally, the early and late stages of pore-filling do not correlate with the alterations in the Porod slope (Fig. S4, ESI†), indicating that pore-filling dynamics depend on more complex factors such as the local density and structure of the pores (*i.e.*, shape and entrance), which are difficult to quantify at this point. This behavior can be explained by the inhomogeneous nature of HC, where variations in local packing densities affect this complex process by altering the energy barrier for quasimetallic clustering.

In the early plateau region, the progressive insertion or confinement of sodium ions may lead to local accumulation and enhanced electrostatic repulsion, which has been suggested as a cause of decreased sodium ion mobility in prior studies.^30,47^ As the applied potential increases, ions interact with the pore surfaces and recombine with the stored counter-electrons, transitioning into a near-neutral charge state (Na^+^ → Na^*α*^, where *α* < 1) during the early plateau, forming a monolayer film. When this monolayer is saturated, storage transits to the late plateau behavior where multi-layer structures are energetically accessed. This progression can be described analogously using the Brunauer–Emmett–Teller (BET) theory of gas physisorption, where increasing gas pressure drives the transition from monolayer to multilayer adsorption. During the late plateau, we propose that sodium ions form quasi-metallic clusters, reducing local charge repulsion and enabling denser multilayer-like packing, which corresponds to the observed increase in the diffusion coefficient.^47^ This process resembles again the saturation of adsorption sites in BET theory, where multilayer adsorption culminates in pore condensation. In the sodium-ion system, multilayer-like clustering enables denser configurations, allowing more ions to occupy the internal pores. This dynamic continues until the slit- and micro-pores are filled. Beyond this point, sodium metal plating at the outside of the carbon pore system and particles initiates below 0 V (*vs.* Na^+^/Na).^30^ Therefore, the electrode is also investigated beyond the nucleation overpotential during the sodium metal plating process. During this loading phase, as the micropores are already filled, we observe negligible to minor changes in Δ*ρ̃*, as expected (Fig. S9, ESI†). This indicates that sodium plating occurs primarily at the outer surface of the electrode, as anticipated before.^13^ Importantly, while sodium clusters form within confined micropores, surface-plated sodium does not readily convert into stable clusters, as the formation of clusters requires confinement within nanoscale voids.^9^

### 2.3. *Operando* Raman and WAXS to reveal adsorption and intercalation

Typical hard carbons, characterized by their disordered structure, exhibit regions with altered electronic properties. These regions can attract and hold sodium ions more effectively due to localized electronic interactions, making adsorption more favorable. This adsorption of sodium ions on active carbon sites is anticipated to impact the Raman signature of the material, potentially shifting the position or changing the intensity of the D- and G-bands.^29,47–50^ Therefore, to explore the changes occurring in the C–C bonds of HC during battery cycling, *operando* Raman spectroscopy measurement was employed. The experimental details and data processing regarding the *operando* Raman spectroscopy can be found in Supplementary Note 3 (ESI†).

**Fig. 3:**
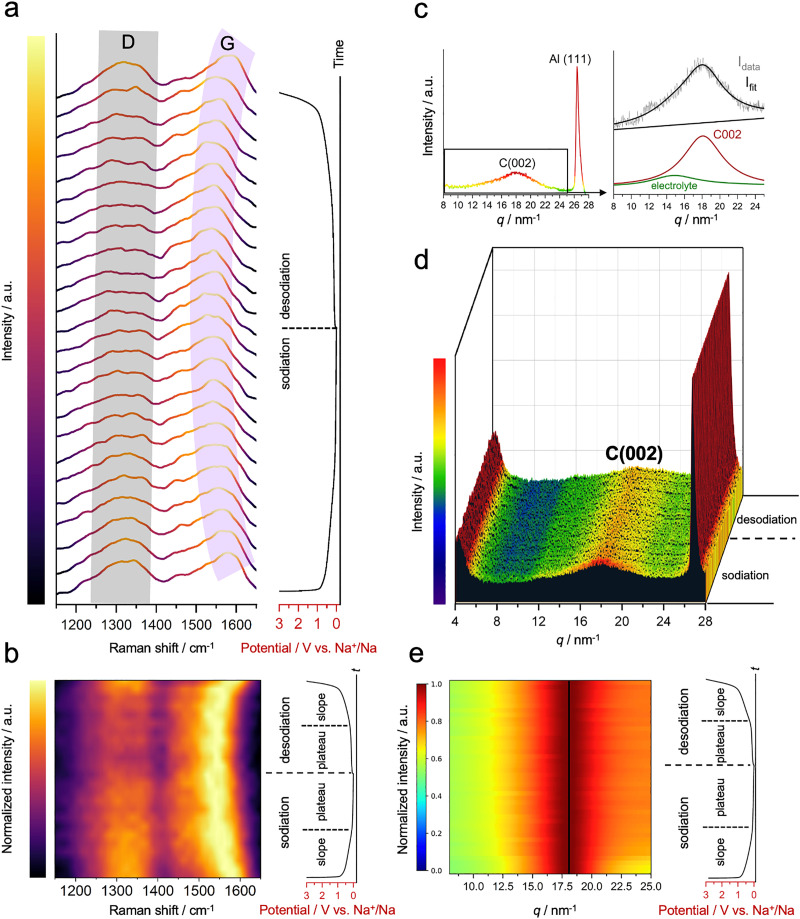
(a) Raman spectra of the HC during sodiation and desodiation, revealing slight intensity changes in the D-band and a sharp shift in the G-band. (b) A heat map with normalized intensity visualizes the pronounced shift in the G-band during sodiation and desodiation. (c) *Operando* WAXS pattern, showing the C(002) peak of hard carbon alongside contributions from the Al current collector and a broad, insignificant background from the electrolyte. The C(002) peak is resolved and isolated *via* peak fitting, with no overlap from electrolyte contributions. (d) WAXS patterns during sodiation and desodiation, revealing no shift in the C(002) but a slight decrease in intensity at the beginning of the electrochemical process. (e) Normalized heat map of the deconvoluted C(002) peak visualizes no pronounced shift during the electrochemical processes.

The first observation reveals a slight decrease in the intensity of the non-normalized data for the D-band during sodiation, indicating the change in the A_1g_ symmetries (Fig. 3a and Fig. S13, ESI†), as sodium ions interact with defect sites by partially saturating or modifying the electronic states associated with these defects. This interaction modifies the symmetry-breaking effects and reduces defect-induced scattering within the graphene Brillouin zone, which manifests as a subtle decrease in the D-band signal.^29,47,48^ However, this information does not stand out compared to the sharp downshift observed for the G-band. During sodiation, we observe a downshift of the G-band peak from ∼1580 to ∼1540 cm^−1^, occurring in the slope region (Fig. 3b). This has been previously explained by the charge transfer between alkali ions and the π* antibonding states of the carbon structure, changing the length of C–C bonds due to electron–phonon coupling.^29,47^ While such evolution is often interpreted as evidence of sodium insertion, similar downshifts can also arise from ionic Na–C interactions during surface adsorption, which cause electron doping and modulate the Fermi level of the carbon host.^51^ Intercalation in combination with adsorption has been noted to produce a distinct peak splitting in the Raman spectra due to adjacent intercalated graphene layers.^9,29^ However, such splitting is not observed here. Given this, we propose that the observed G-band shift in the slope region is predominantly attributed to surface adsorption. This interpretation is also based on the large capacity contribution from the slope region arising from the surface-driven processes, as proved by electrochemical characterization. If the shift was due to intercalation, the corresponding lattice strain would be recorded. To further investigate this behaviour and validate this hypothesis, we conducted *operando* WAXS measurements. The carbon covers a broad range of adsorption sites with varying free energies due to its uneven nature, and is reflected in deviation from the linearity of the capacitive region. At the plateau, the position of the G-band remains rather constant, where the faradaic processes dominate. Upon desodiation, the Raman bands follow a reverse trend: the relative intensity of the D-band increases, and the position of the G-band gradually returns to its initial state, reflecting the reversibility of the adsorption processes (Fig. 3b and Fig. S13, ESI†).

In principle, alkali ion intercalation may induce strain in the stacking, leading to changes in local density, increased *d*-spacing, and a shift of diffraction peaks to lower *q* (or 2*θ*) values.^49,50^ For example, lithium intercalation into a graphitic structure to form LiC_6_ results in a downshift of the C(002) peak by more than a degree, caused by the strain within the graphitic planes.^52,53^ Thereby, *operando* WAXS is employed to monitor the possible intercalation happening in the hard carbon. The peak of Al(111) of the current collector (∼26.5 nm^−1^) is visible, along with the broad C(002) peak (∼18 nm^−1^) (Fig. 3c); the latter becomes more pronounced after data refinement. The experimental details and data processing regarding the *operando* WAXS can be found in Supplementary Note 4 (ESI†). Here, it is worth mentioning that the HC material is directly cold-pressed onto the aluminum current collector to eliminate the contributions from the conductive carbon additive and binder, which might have similar scattering profiles to the HC.

At the onset of the sodiation process, we observe a slight decrease in the intensity of the C(002) peak, attributed to non-structural artifacts, such as electrolyte diffusion, causing variations in the effective scattering length density and impacting the intensity of the observed peaks (Fig. 3d). While some studies associate this with sodium insertion processes at the slope region,^54^ we do not observe any reversibility in intensity changes during desodiation. Thus, integrating it with the already complex electrochemical processes is challenging to justify at this scale. Additionally, during both sodiation and desodiation, the C(002) peak does not shift to lower *q* values, thereby ruling out the insertion in the pseudo-graphitic layers (Fig. 3e). Recent studies have reported the absence of classical sodium intercalation in hard carbon anodes during both the sloping and plateau regions, as evidenced by the lack of significant changes in structural measurements.^9,20,55^ Consistent with these findings, our results further support a sodium storage mechanism dominated by surface adsorption, monolayer accumulation, and pore filling, without notable interlayer expansion of graphitic domains. The critical information here is that ion diffusion does not occur evenly in the graphitic regions; instead, it happens in the amorphous areas with larger periodicity or defects, such as edges. This implies that sodium intercalation is unlikely to play a critical role in the overall sodium storage mechanism.

The deposition of near-zero valent sodium initiates at the early plateau stage. It happens primarily in the regions where the interface energy is favorable to initiate surface wetting.^9^ This results in a uniform monolayer of quasimetallic sodium, contributing 27% of the overall capacity (early plateau). The gradual decrease in the diffusion coefficient reflects the increasing saturation of these surfaces. The final stage involves the reduction of additional sodium atop the initial monolayer, forming a multilayer-like cluster within the micro- and slit-pores. The formation of these clusters reduces the effective charge repulsion between sodium ions by lowering the local positive charge density. As a result, the migration of additional sodium ions into the pores becomes energetically more favorable, facilitating improved ion mobility. This transition leads to a relative stabilization and an increase in the diffusion coefficient. The multilayer-like deposition process contributes about 31% of the overall capacity (late plateau). *Operando* SAXS measurements support this mechanism, showing a sharp decrease in electron density differences due to the significant increase in sodium volume ratio within the pores.

**Fig. 4:**
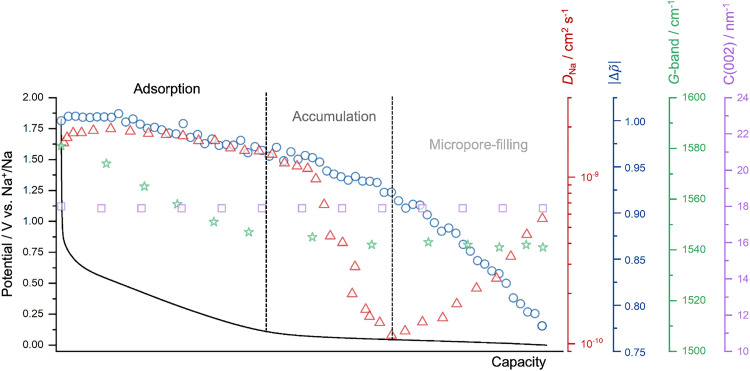
The summary of the main findings regarding the *operando* investigations, along with a galvanostatic sodiation curve, *i.e.*, the trends in sodium-ion diffusion coefficients obtained from GITT (in red, plotted against capacity rather than potential, as in Fig. 1g, for comparative analysis), the change in electron density differences (Δ*ρ̃)* of micropores (in blue), the G-band peak of the carbon (in green), and the scattering vector (*q*) of the C(002) peak (in purple).

It is worth noting that material modifications such as heteroatom doping and electrolyte composition can influence sodium storage behavior. For instance, nitrogen doping introduces additional defect sites and heteroatom functionalities, which can facilitate sodium accumulation and pore filling, leading to an increased plateau capacity.^56^ Similarly, the nature of the electrolyte affects the sodium ion solvation environment: ether-based electrolytes, with weaker solvation and lower desolvation barriers, enhance ion transport and favor more efficient pore-filling processes, while ester-based electrolytes can hinder these steps due to stronger solvation energy.^4,57,58^ Nevertheless, these variations primarily modulate the energetics and kinetics of each storage stage without altering the overall sequential mechanism. Based on those, we propose the adsorption-accumulation-filling model as a more accurate and contemporary framework for describing the sodium storage mechanism in hard carbons (Fig. 4).

## 3. Conclusions

Combining electrochemical characterizations with *operando* SAXS, WAXS, and Raman spectroscopy provides a refined understanding of sodium storage mechanisms in hard carbons. The proposed three-stage model identifies a capacitive mechanism occurring in the slope region, transitioning through an early plateau with a decrease in sodium-ion mobility, and culminating in a dominant micropore-filling mechanism during the late plateau, as indicated by *operando* SAXS. *Operando* Raman spectroscopy illustrates changes in the G-band position due to strong ionic Na–C interactions, while *operando* WAXS highlights the absence of visible intercalation evidences. We believe that the current model will serve as a basis for designing electrode materials with superior performance by fine-tuning the porous structure of hard carbons and correlating it to the relevant electrochemical energy storage steps.

## Conflicts of interest

There are no conflicts to declare.

## Supplementary Material

EE-018-D4EE06029F-s001

## Data Availability

The data supporting this article have been included as part of the ESI.†
