# Supplementary material for: An enhanced three-stage model for sodium storage in hard carbons
Source: Energy Environ Sci. 2025 Jun 5;18(16):7859–68. doi: 10.1039/d4ee06029f (PMC12138748; doi:10.1039/d4ee06029f)
Supplement: EE-018-D4EE06029F-s001 [file EE-018-D4EE06029F-s001.pdf]

Supplementary Information (SI) for Energy & Environmental Science.
This journal is © The Royal Society of Chemistry 2025

# Supplementary Information

## An enhanced three-stage model for sodium storage in hard carbons

*Enis Oğuzhan Eren,*[a] Evgeny Senokos,[a] Ernesto Scoppola,[b] Zihan Song,[a,c] Markus Antonietti,[a] and Paolo Giusto*[a]*

[a] *Department of Colloid Chemistry, Max Planck Institute of Colloids and Interfaces, Potsdam 14476, Germany*

[b] *Department of Biomaterials, Max Planck Institute of Colloids and Interfaces, Potsdam 14476, Germany*

[c] *Department of Engineering Science, University of Oxford, Oxford OX1 3PJ, United Kingdom*

**E-mail; enis.eren@mpikg.mpg.de; paolo.giusto@mpikg.mpg.de*

**Supplementary Note 1**

**Synthesis of hard carbon and electrode preparation.** In our previous study, we detailed an extensive procedure for the synthesis and characterization of HC.[(1)] To summarize, we combined graphitic carbon nitride (g-CN, 500 mg) with 3,4-ethylenedioxythiophene (EDOT, 97%, Sigma-Aldrich, 10 mL) in a vial and subjected it to ultrasonication for approximately 10 minutes. Subsequently, the vial was exposed to a 50 W visible light source with continuous stirring for 24 hours at room temperature to initiate photopolymerization. The resulting viscous honey-like fluid, oligo-EDOT, was separated from g-CN through filtration. For pyrolysis, oligo-EDOT was transferred to an ashing furnace (Nabertherm, Germany) and heated to 1000°C at a rate of 3.15 K $min^{-1}$ under a nitrogen atmosphere, maintaining this temperature for 2 hours. The resulting material, a thin, crispy film exhibiting a metallic sheen, was then finely ground into a powder.

Electrodes were produced by blending HC with conductive carbon black (Super-P, Alfa Aesar) and polyvinylidene difluoride (PVDF, Kynar HSV-900) binder at a ratio of 8:1:1 by weight. PVDF was dissolved in *N*-methylpyrrolidone (NMP, Sigma-Aldrich). The resulting slurry was applied onto aluminum foil (15 µm) using a film applicator (mtv messtechnik, Germany) and dried overnight in a vacuum oven (Thermo Fisher, USA) at 80 °C. The active material mass in the electrodes was approximately 1.0 mg $cm^{-2}$. All electrochemical assessments were performed using three-electrode Swagelok-type cells on a Biologic MPG-2 instrument (France). Swagelok-type cells were assembled in an argon-filled glovebox (MBRAUN, Germany) with $H_2O$ and $O_2$ levels below 0.1 ppm. The electrolyte used was 1M $NaPF_6$ in ethylene carbonate (EC)/ethyl methyl carbonate (EMC) (3:7 by volume, E-Lyte GmbH, 200 µL), glass fibers (Whatman GF/C) used as separators, and a thin slice of sodium metal (99.5%, Sigma-Aldrich) served as both the counter and reference electrode.

**Electrochemical characterizations.** The galvanostatic charge-discharge curves of the half-cells were recorded within the potential window of 0-2.5 V (vs. $Na^+$/Na). Before measurements, the half-cells underwent a six-hour resting period. The rate performance assessment of the electrode was conducted at 30 mA $g^{-1}$ and 300 mA $g^{-1}$.

Cyclic voltammetry (CV) measurements were performed at scan rates of 0.05, 0.1, 0.2, 0.5, and 1.0 mV $s^{-1}$ across the potential range of 0-2.5 V (vs. $Na^+$/Na). Electrochemical impedance spectroscopy (EIS) was performed with an AC perturbation of 10 mV in the frequency range of 0.1 Hz to 20 kHz. EIS spectra

were analyzed using DRT analysis to deconvolute the overlapping electrochemical processes based on their characteristic time constants. Measurements were carried out at various fixed potentials during sodiation to evaluate changes in interfacial resistance and kinetic behavior across different voltage regions.

The galvanostatic intermittent titration technique (GITT) was employed to determine the sodium-ion diffusion coefficients. Throughout the experiments, current pulses (30 mA $g^{-1}$) were applied for 1200 s, followed by measuring relaxation potentials for 3600 s. The diffusion coefficients were subsequently calculated based on these measurements.

We utilized the first-order approximation method suggested by Weppner and Huggins,[2] which is suitable for both spherical and planar geometries.[3] Nevertheless, it's crucial to acknowledge that while this approach is suitable for hard carbons, it relies on certain assumptions.[4-7]

When the step time is relatively shorter than the effective diffusion time, and the transient data are sufficiently large to exclude ohmic and kinetic overpotential, a linear relationship between the potential response and the square root of the step time is observed in the single pulse GITT curve (**Figure S1b**). Consequently, the first-order approximation can be expressed by the following equation (Eq. 1),[4, 5] where the diffusion coefficient derived from this equation is referred to as the effective diffusion coefficient in this study:

$$D_{Na} = \frac{4}{\pi\tau}\left(\frac{m_B V_M}{M_B S}\right)^2\left(\frac{\Delta E_s}{\Delta E_\tau}\right)^2 \qquad (1)$$

Here, $\tau$ represents the pulse duration, $m_B$ and $M_B$ denote the actual and molar mass of the active material, $V_M$ is the molar volume, and $S$ stands for the surface area of the electrodes. The values $\Delta E_s$ (change of the steady-state voltage during a single-step GITT curve) and $\Delta E_\tau$ (change of cell voltage during a constant current pulse) can be derived from the GITT curve of the material (**Figure 1f**).[7, 8] The effective diffusion coefficients of the materials are presented in the manuscript (**Figures 1g, S1c,** and **S1d**).

To quantitatively assess the relative contributions of each storage stage to the overall capacity, we divided the total sodiation process into three regions—slope, early plateau, and late plateau—based on the inflection point in the GCD curve and the minimum diffusion coefficient. The capacity fractions were calculated as follows: the slope contribution corresponds to the capacity accumulated up to the inflection potential in the GCD curve (Eq. 2, from the beginning to the 0.12 V (vs. $Na^+$/Na)); the early plateau is

defined from this inflection point to the potential at which the diffusion coefficient reaches its minimum (Eq. 3); and the late plateau covers the remaining capacity from this point to full sodiation (Eq. 4). The exact formulas yield contributions of approximately 27% for the early plateau and 31% for the late plateau.

$$Slope\ (\%) = \frac{Q_{at\ inflection\ potential}}{Q_{total}} X\ 100 \tag{2}$$

$$Early\ plateau\ (\%) = \frac{Q_{at\ min.\ diffusion\ coefficient} - Q_{at\ inflection\ potential}}{Q_{total}} X\ 100 \tag{3}$$

$$Late\ plateau\ (\%) = \frac{Q_{total} - Q_{at\ min.\ diffusion\ coefficient}}{Q_{total}} X\ 100 \tag{4}$$

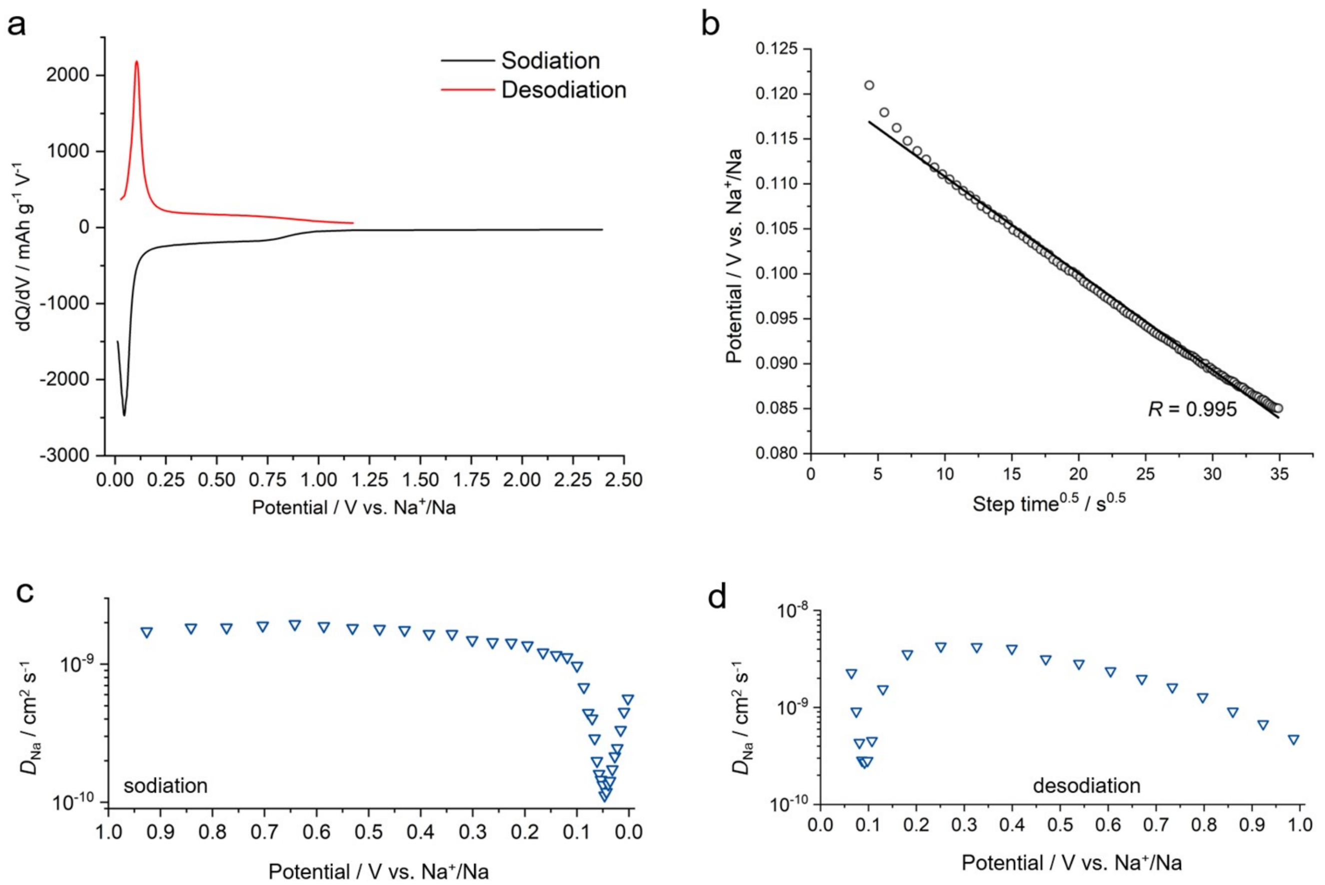


**Figure S1. (a)** d$Q$/d$V$ curve of sodiation and desodiation at 30 mA $g^{-1}$. **(b)** The single-step GITT curve indicates linear relationships between V vs. $s^{0.5}$. Diffusion coefficients for sodiation **(c)** and desodiation **(d)**.

**Supplementary Note 2**

***Operando* small-angle X-ray scattering (SAXS)** measurements were conducted at the µSpot beamline of BESSY-II (Helmholtz-Zentrum Berlin, HZB, Germany).(9) A monochromatic X-ray beam at 18.0 keV was utilized, with a beam size of approximately 30 µm width achieved through a sequence of pinholes. Scattered intensities were detected using a Dectris Eiger 9M detector. Transmission through the sample was determined from an X-ray fluorescence signal recorded by a RAYSPEC Sirius SD-E65133-BE-INC detector equipped with an 8 µm beryllium window positioned in front of a lead beam stop. The primary beam intensity was monitored using an ion chamber, and recorded values were utilized to normalize the scattering signal. With a sample-to-detector distance of approximately 600 mm, a usable $q$-range of about $0.06 < q < 27$ nm$^{-1}$ was achieved. The scattering $q$-range was calibrated using silver behenate, and the resulting intensities were normalized against glassy carbon (NIST SRM3600). Data processing was conducted using in-house Python software based on the pyFAI library.(10) The data reduction process included integrating to obtain 1D scattering curves and subtracting instrumental background, which typically involved measurements from an empty cell or Kapton background. Subsequently, the scattering data underwent correction for transmission and primary beam intensity. During data modeling and fitting, intensity uncertainties and instrumental smearing were considered.

The measurements were performed using a specialized half-cell, as demonstrated in our prior study, featuring Kapton films (8 µm) as transmission windows (**Figure S2**).(1) The configuration consists of the electrode (8:1:1 ratio of active material, conductive carbon, and PVDF binder) on an Al current collector, separator, and a thin sodium foil counter/reference electrode, resembling a two-electrode sandwich cell setup. Before measurements, the cell underwent a careful leak test and pre-cycling to ensure stable performance. For on-site operando SAXS measurements, a Gamry Interface 1010 (USA) portable potentiostat was utilized. Sodiation and desodiation processes were conducted at a current density of 30 mA g$^{-1}$.

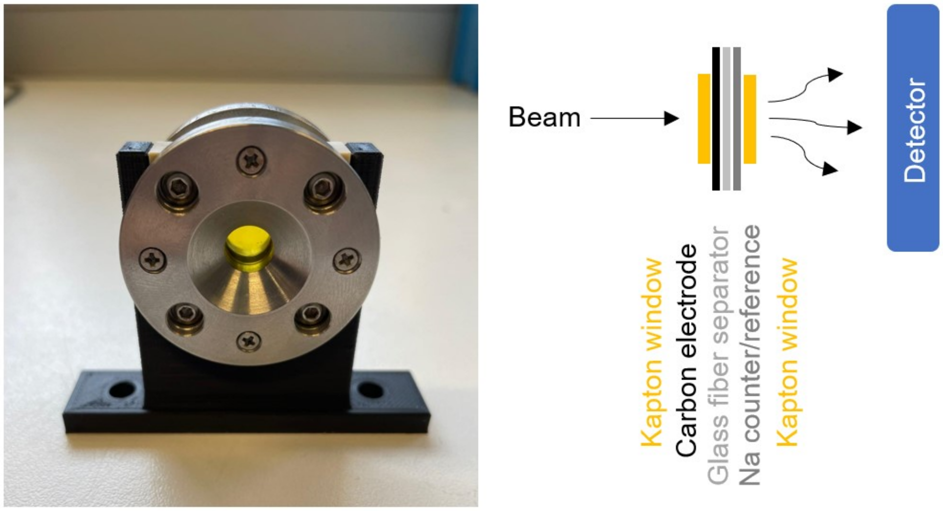


**Figure S2.** A stainless-steel custom *operando* electrochemical cell with transmission windows.

To address the inhomogeneity arising from the amorphous nature of the hard carbon, the sample electrode was mapped at 16 distinct regions to assess consistency in structural changes. However, six regions were excluded due to certain errors, leaving a total of ten spots presented in the manuscript (designated as Region 1 to Region 10) to illustrate the consistency. Scattering data were collected with a time resolution of 4 seconds during both the sodiation and desodiation processes.

The intensity of scattering, denoted as $I(q)$, is recorded based on the momentum transfer $q = {}^{4\pi}/_{\lambda} sin(\theta)$, where λ represents the wavelength of photons and θ is the scattering angle. The scattering signal can be expressed in terms of the difference in scattering length density (SLD), denoted as $\Delta\rho$, between particles or objects and the surrounding medium.

$$I(q) = \phi \Delta\rho^2 P(q) S(q) \tag{5}$$

Here, $\phi$ represents a scaling constant that is directly proportional to the particle volume fraction. $P(q)$ denotes the form factor function, which characterizes the shape of the particles, while $S(q)$ describes their spatial distribution or correlation. Past studies have demonstrated the effectiveness of fractal-like models in explaining small-angle scattering from carbonaceous materials.(11) Within this context, we analyzed our time-dependent data by considering the contribution to the scattering signal from the carbonaceous structure, denoted as $I_{Porod}$, and from the micropores, labeled as $I_{mp}$.

$$I(q,t) = \alpha \cdot [I_{Porod}(q,t) + I_{mp}(q,t)] + b(t) \tag{6}$$

The symbol $\alpha$ represents an arbitrary scaling factor, where the data are not scaled in absolute units, and $b(t)$ represents a background term independent of $q$. $I_{Porod}(q,t)$ corresponds to Porod's law, describing scattering by sharp interfaces resulting from the macroscopic surface of the carbon electrode. It can be expressed as shown in Eq. 7.

$$I_{Porod}(q,t) = 2\pi \cdot \phi_0(t) \cdot \Delta\rho_0^2(t) \cdot P(t) \cdot q^{s(t)} \tag{7}$$

Here, $\phi_0$ represents the volume fraction of the carbonaceous material, $\Delta\rho_0$ stands for the SLD difference with the surrounding environment (such as the Al current collector and electrolyte), $P$ denotes the specific surface of the carbon, and $s$ represents the slope in the double logarithmic plot (referred to as the Porod slope) of the small-angle signal for $q$ values less than 0.5 nm$^{-1}$. SAXS of pseudo-graphitic carbons has a typical Porod slope value of approximately -4, as detailed by Saurel et al.[11] The contribution of micropore scattering, denoted as $I_{mp}(q,t)$, can be expressed as follows:

$$I_{mp}(q,t) = \rho_{struc}^{-1} \cdot \phi_1(t) \cdot \Delta\rho_1^2(t) \cdot I_1(q,t) \cdot S_1(q,t) \tag{8}$$

where $\phi_1$ is the volume fraction of micropores, $\Delta\rho_1$ is their SLD difference with the carbon matrix, and $\rho_{struc}$ is the structural density, which is calculated using the Eq. 9.

$$\rho_{struc} = \rho_{graphite} \frac{d_{002}}{d_{002}^{graphite}} \left( \frac{d_{100}}{d_{100}^{graphite}} \right)^2 \tag{9}$$

In the given context, $\rho_{graphite}$ represents the structural density of graphite, set at 2.26 g cm$^{-3}$. $d_{002}$ and $d_{002}^{graphite}$ denote the interlayer distances of the sample and crystalline graphite, respectively, while $d_{100}$ and $d_{100}^{graphite}$ refer to the in-plane distances of the sample and crystalline graphite. The fractal structure factor, denoted as $S_1(q,t)$, and the form factor, labeled as $I_1(q,t)$, can be expressed as follows:

$$S_1(q,t) = 1 + \frac{D(t)}{r(t)^{D(t)}} \xi(t)^{D(t)} \frac{D(t)\Gamma(D(t) - 1)}{(1 + (q\xi(t))^2)^{0.5(D(t) - 1)}} \frac{\sin((D(t) - 1)\tan^{-1}(q\xi(t)))}{q\xi(t)} \tag{10}$$

$$I_1(q,t) = \exp\left(-\frac{qr(t)^2}{5}\right) + \frac{9k}{2(qr(t))^4} \left[\mathrm{erf}\left(\frac{qr(t)}{\sqrt{10}}\right)\right]^{12} \tag{11}$$

Here, $D$ represents the fractal dimension, $\xi$ stands for the correlation length, $r$ denotes the radius of pores ($d/2$), and $k$ is a factor associated with the shape of pores. A value of $k$=1 was chosen, assuming spherical micropores.

While the mentioned model has been demonstrated to describe carbonaceous materials effectively, it relies on several parameters. However, during our experiment, scattering patterns were consistently obtained from the same areas. Consequently, structural parameters such as $\phi, P, D, \xi$, and $d$ were determined for each spot in the initial data frame and kept constant for subsequent frames. With these assumptions, two fitting models were applied. Initially, the Porod slope was allowed to vary across frames, yielding values consistently close to -4 for the measured spots (**Figure S4**). Therefore, in the second approach, the function $s(t)$ was fixed at -4 for all fits to prevent parameter interference. Further details, including the structurally relevant best-fit parameter values for $D, \xi$, and $d$, are provided in **Table S1**. The same constraint was applied to the scaling parameter $\alpha$ in Eq. 5. Exemplary fits are illustrated in **Figure S5**. Initial examination indicates a high fitting quality. This, coupled with the Porod slope values reported in **Figure S4**, suggests that it is reasonable to assume the material exhibits characteristic sharp interfaces.

While quantifying the actual closed pore volume would be valuable, extracting absolute pore volumes from operando SAXS data is not feasible in our case due to the inability to reduce the scattering intensity into absolute units ($cm^{-1}$) without precise knowledge of the local electrode thickness. Given slight spatial inhomogeneities, dynamic structural changes, and the limited beam size (30 microns), accurately determining absolute thickness at the measurement location is not possible. Moreover, absolute scaling based on bulk material characterizations (i.e., bulk carbon powder measured in capillaries) would not directly translate to the operando environment. Therefore, we focus on the relative changes in electron density differences ($\Delta\tilde{\rho}$) to robustly capture the progression of pore filling during sodium storage.

**Table S1.** Calculated structural parameters from the first data frame for ten different measuring spots.

| Measurement areas | $d$ / nm | $\xi$ / nm | $D$ |
|---|---|---|---|
| **Region 1** | 1.86 | 2.75 | 1.00 |
| **Region 2** | 1.86 | 4.35 | 1.44 |
| **Region 3** | 1.78 | 1.95 | 1.29 |
| **Region 4** | 1.86 | 5.95 | 1.05 |
| **Region 5** | 1.82 | 2.85 | 1.58 |
| **Region 6** | 1.80 | 2.00 | 1.48 |
| **Region 7** | 1.88 | 1.90 | 1.21 |
| **Region 8** | 1.72 | 4.05 | 1.58 |
| **Region 9** | 1.82 | 3.35 | 1.24 |
| **Region 10** | 1.84 | 1.57 | 1.51 |

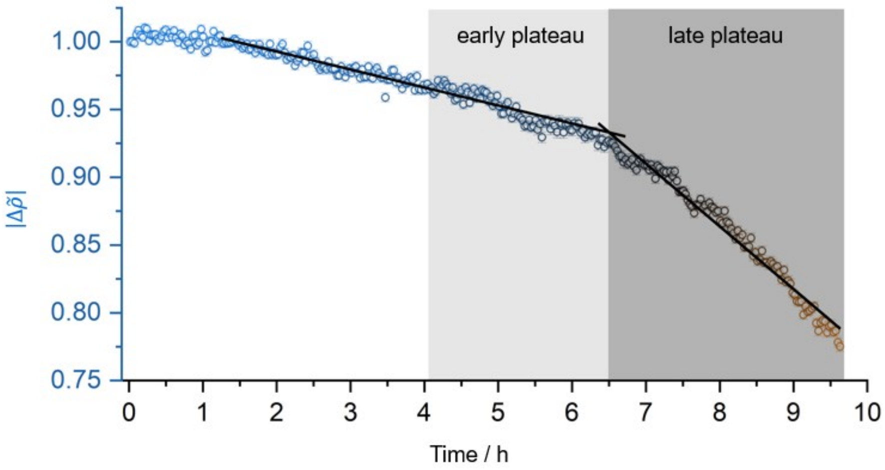


**Figure S3.** Two linear fits, corresponding to two different regions, illustrate the cross-section that follows the early and late plateau trends observed in sodium-ion diffusion coefficients from GITT.

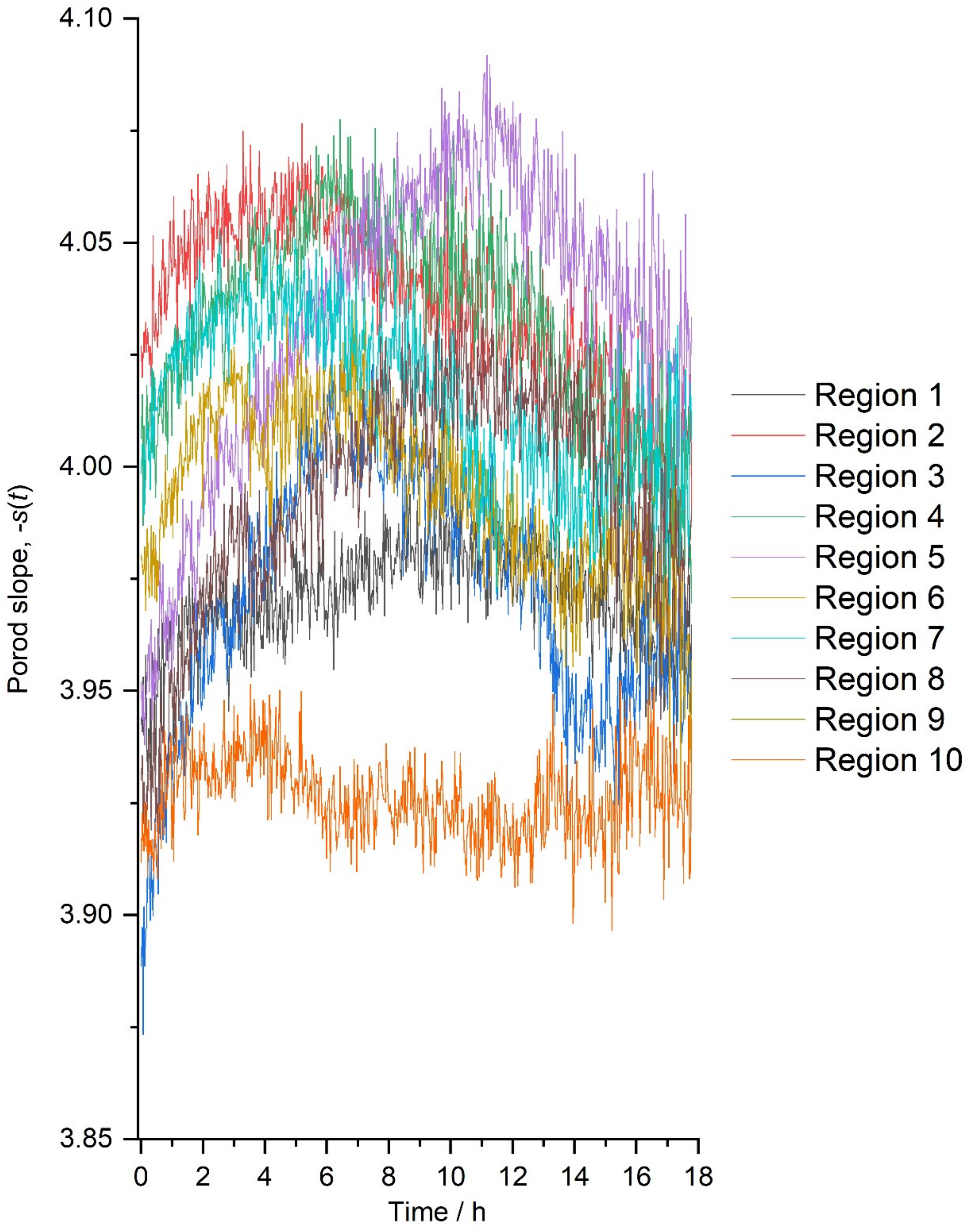


**Figure S4.** The Porod slope observed during both sodiation and desodiation on the log-log plot for the small-angle signal across the ten measurement locations, indicating a value close to 4 with minimal fluctuation.

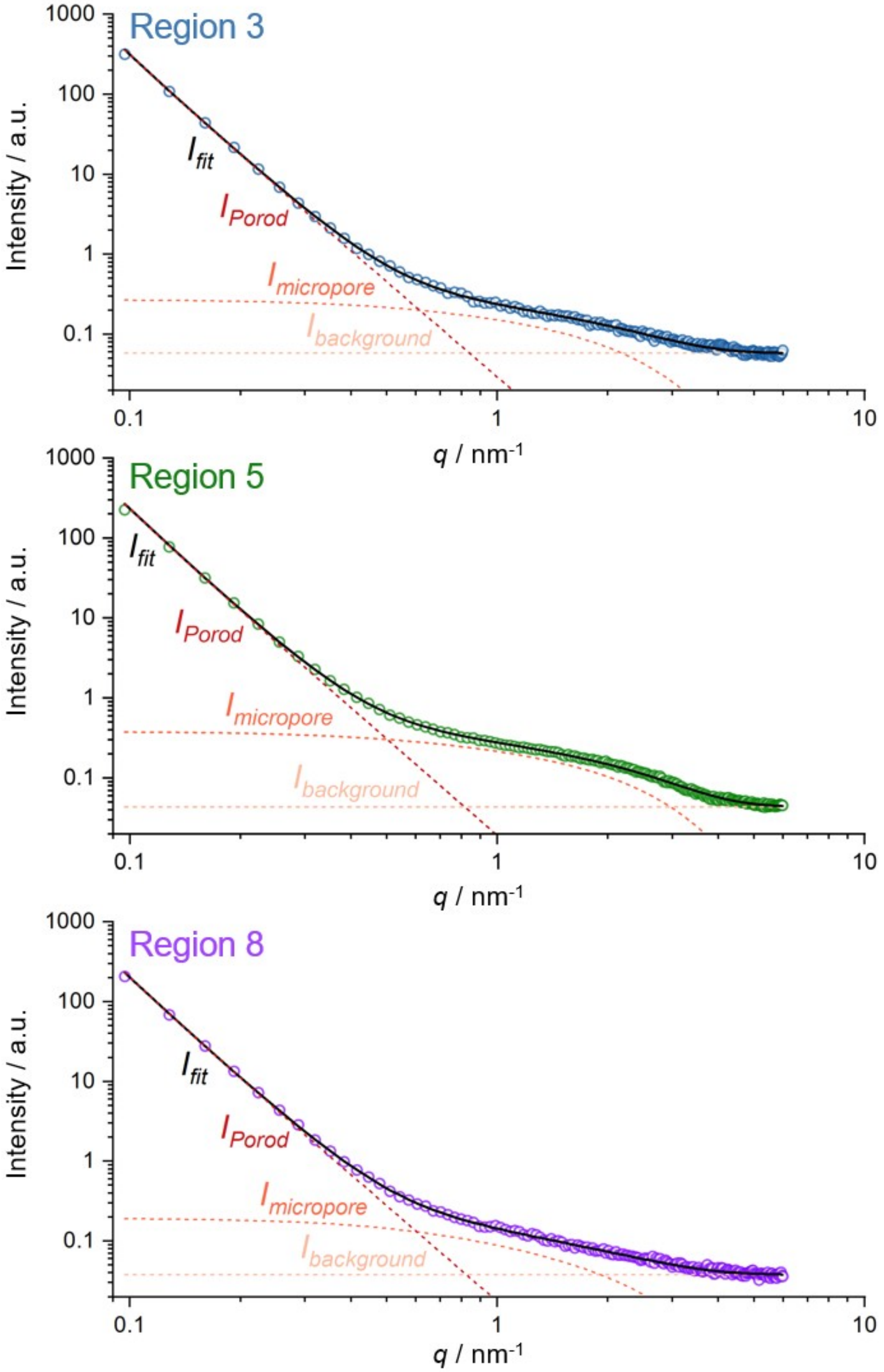


**Figure S5.** Data and fits from three randomly chosen regions were collected at the same timestamps, demonstrating the precision of the fitting model.

The constraints previously mentioned were selected under the assumption of negligible structural variations during the sodiation-desodiation. This facilitates the variations in the SLD difference, enabling the elucidation of the pore-filling mechanism and its correlation with electrochemical changes during operation. By utilizing Eq. 7 and 8, we distinguish variations in the SLD difference into two distinct components: $\Delta\rho_0$ and $\Delta\rho_1$. Specifically, $\Delta\rho_0$ represents the SLD difference between the carbon structure, encompassing features like locally arranged layers and micropores, and the surrounding environment (e.g., Al current collector and electrolyte solution). Thus, an increase in $\Delta\rho_0$ is anticipated during processes such as adsorption and diffusion of sodium ions within the carbon structure and micropores, without the ability to deconvolute these processes at this stage. Meanwhile, the differentiation between micropores and bulk structural features of the carbon material in terms of SLD difference is explained by $\Delta\rho_1$, identified as the critical parameter for investigating the micropore-filling mechanism. A deeper insight into micropore-filling kinetics is achieved by introducing a new parameter denoted as $\Delta\tilde{\rho}$, defined as the normalized ratio between $\Delta\rho_1$ and $\Delta\rho_0$ (Eq. 12). In this context, $\Delta\tilde{\rho}$ measures the relative change in the micropores compared to the overall carbon structure.

$$\Delta\tilde{\rho}(t) = \frac{\Delta\rho_1(t)\Delta\rho_0(t=0)}{\Delta\rho_0(t)\Delta\rho_1(t=0)} \tag{12}$$

As illustrated in **Figures S6**, **S7**, and **S8**, $\Delta\rho_0$ steadily increases throughout the electrochemical cycle. This is attributed to irreversible processes at the interfaces, such as electrolyte decomposition, resulting in trapped electrons that create contrast. **Figure S10** displays the $\Delta\tilde{\rho}$ variation across ten different regions on the electrode during the sodiation process. The smoothed map of this data (using an adjacent average of 30 points) is presented in the main manuscript as **Figure 2e**.

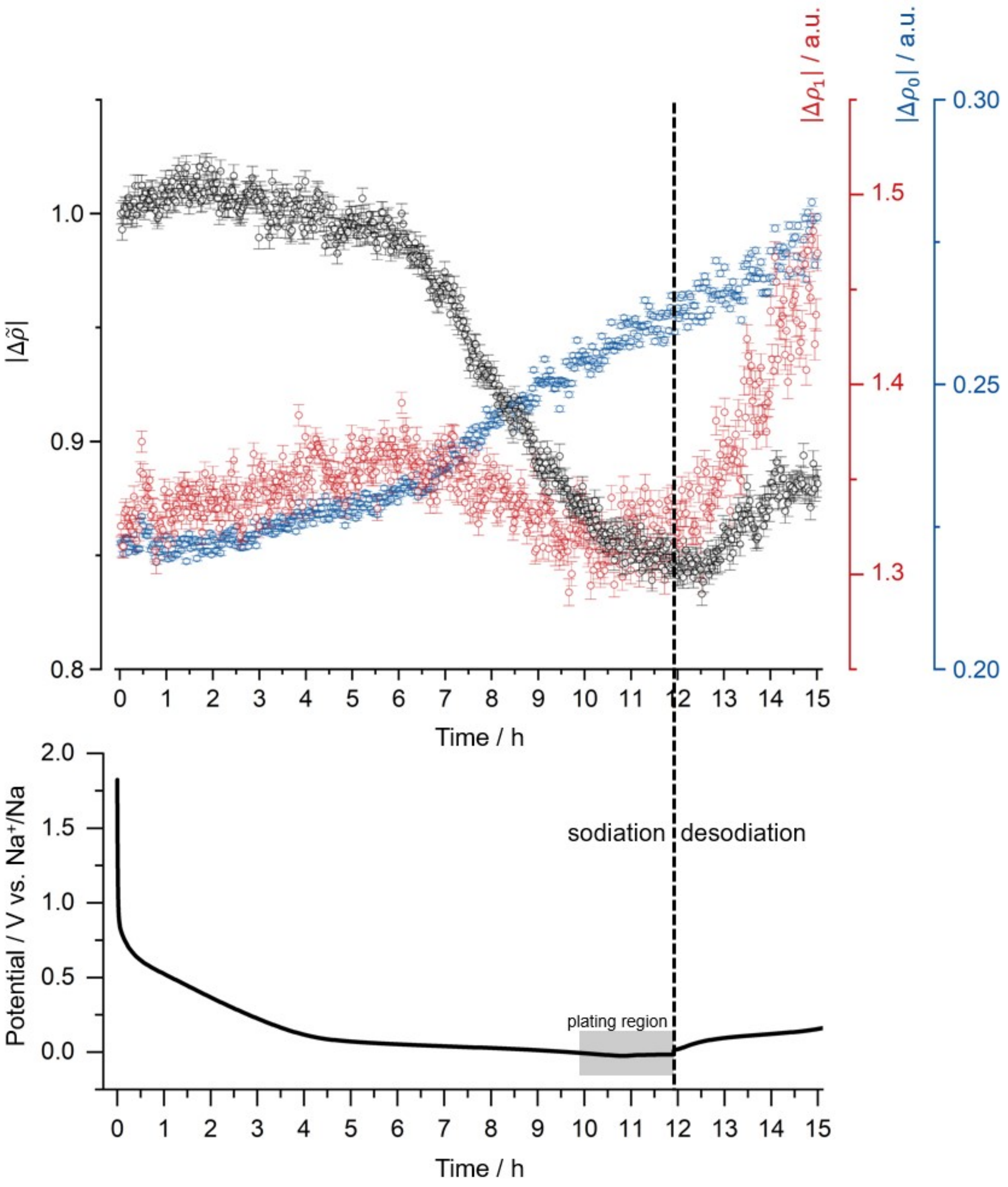


**Figure S6.** Changes in $\Delta\rho_0(t)$, $\Delta\rho_1(t)$, and $\Delta\tilde{\rho}(t)$ throughout the sodiation and desodiation processes in Region 3. The overpotential sodium deposition is shown by a transparent gray segment on the GCD curve. Uncertainties are depicted by propagating standard errors derived from the estimated parameters.

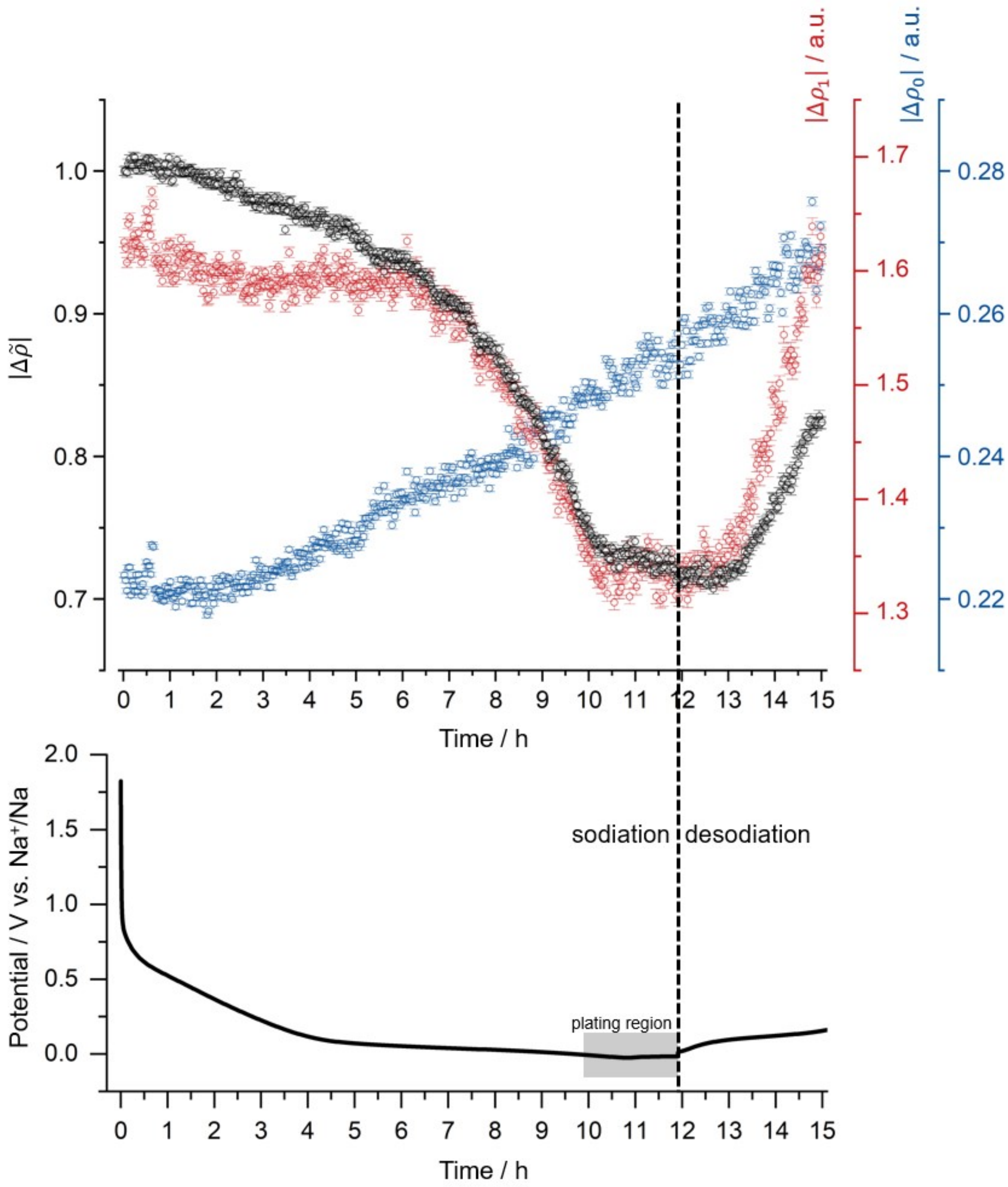


**Figure S7.** Changes in $\Delta\rho_0(t)$, $\Delta\rho_1(t)$, and $\Delta\tilde{\rho}(t)$ throughout the sodiation and desodiation processes in Region 5. The overpotential sodium deposition is shown by a transparent gray segment on the GCD curve. Uncertainties are depicted by propagating standard errors derived from the estimated parameters.

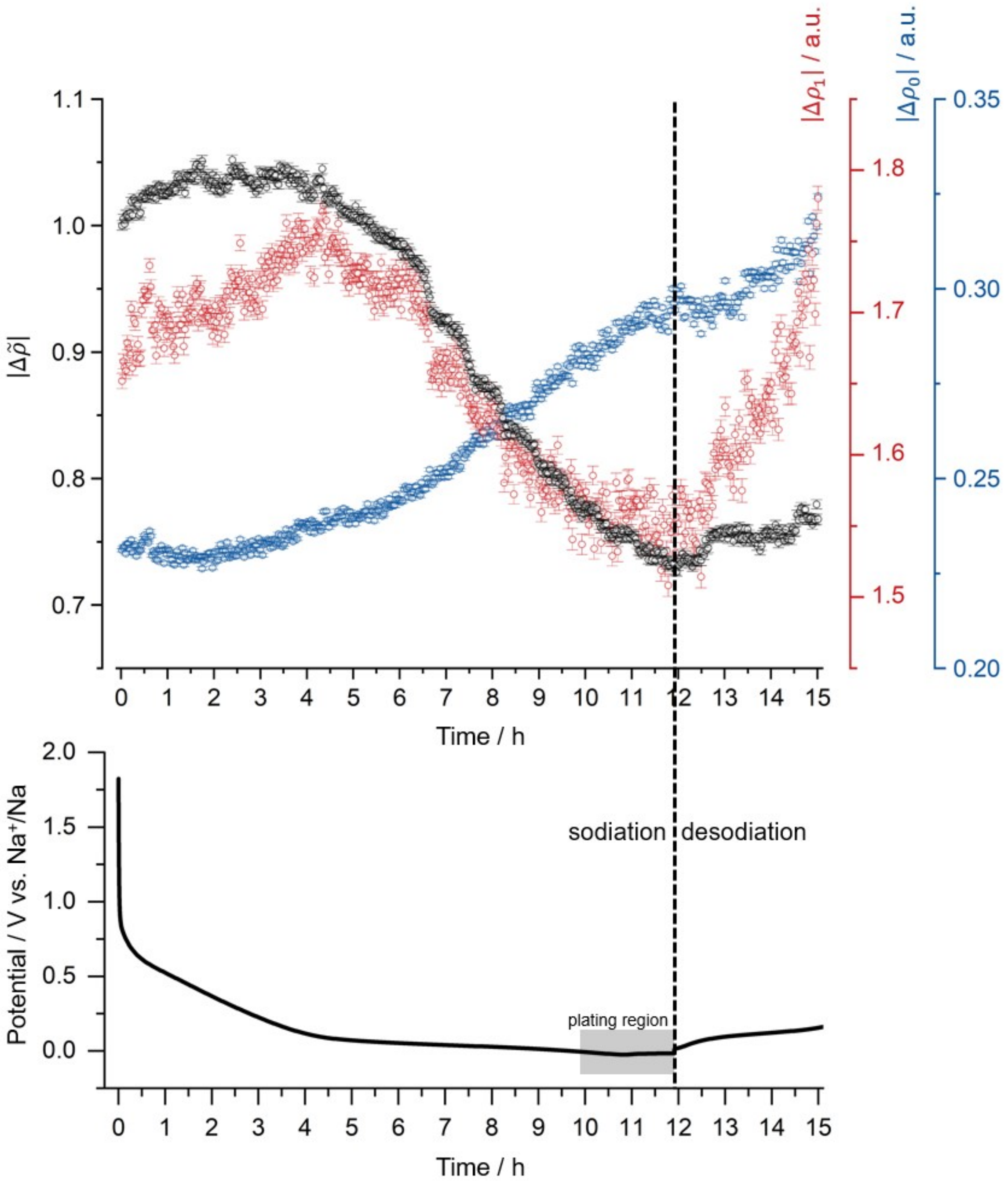


**Figure S8.** Changes in $\Delta\rho_0(t)$, $\Delta\rho_1(t)$, and $\Delta\tilde{\rho}(t)$ throughout the sodiation and desodiation processes in Region 8. The overpotential sodium deposition is shown by a transparent gray segment on the GCD curve. Uncertainties are depicted by propagating standard errors derived from the estimated parameters.

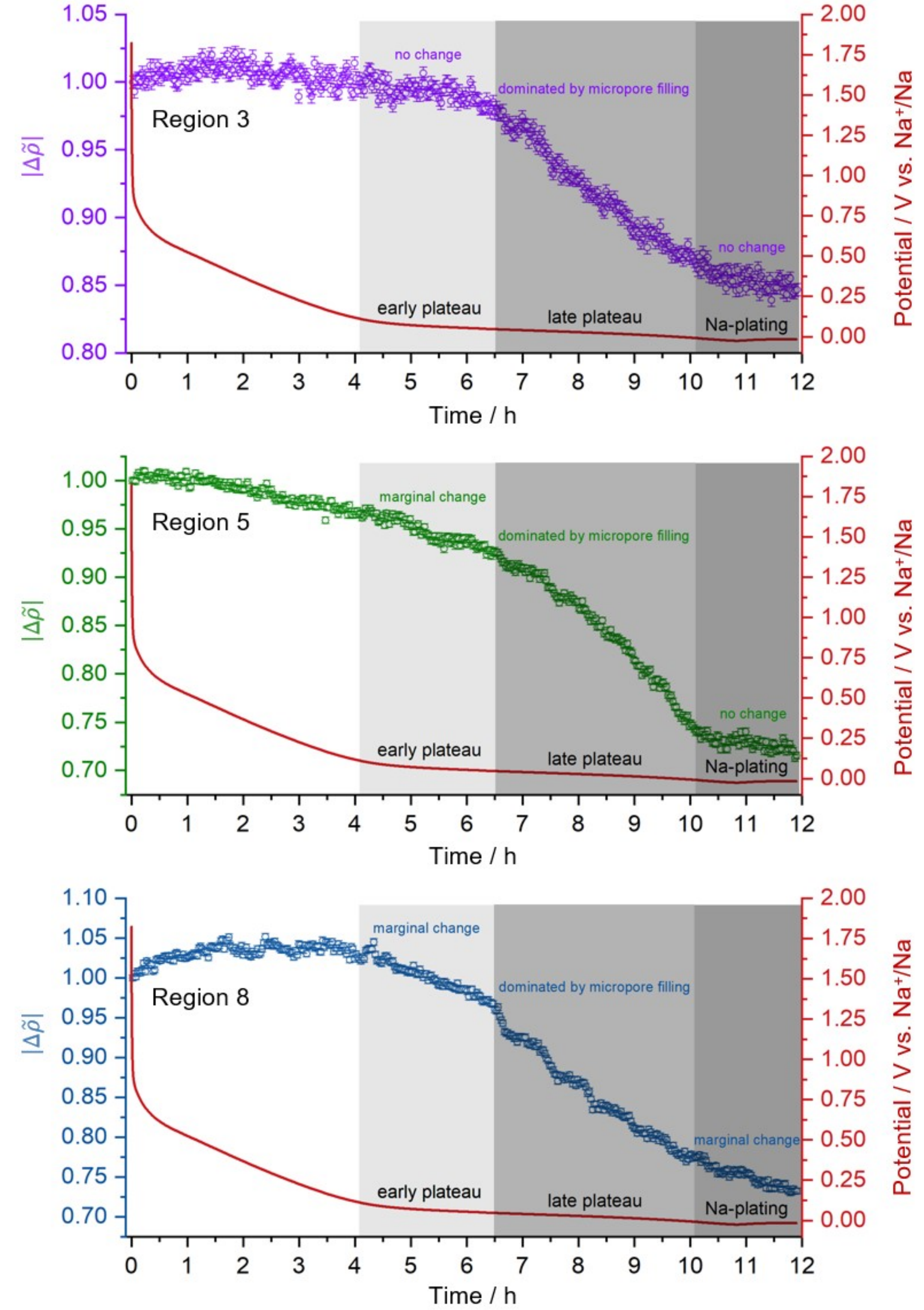


**Figure S9.** The full scale of the sodiation part in Regions 3, 5, and 8, including the sodium plating region, eventually has little to no effect on the micropore-filling mechanism.

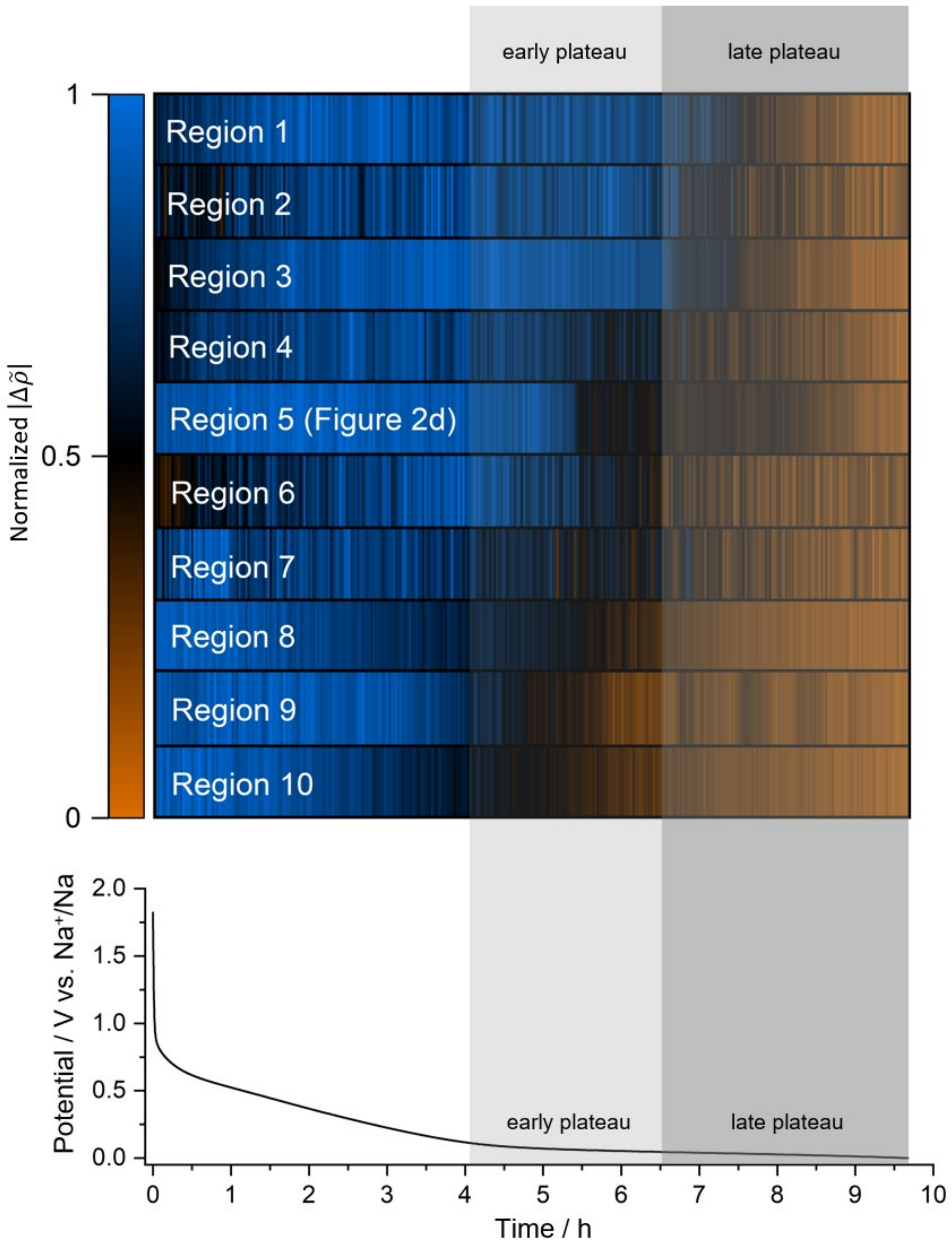


**Figure S10.** A heat map visualizes ten different regions in the same electrode for the reproducibility of the analysis. Regions have a similar trend in normalized $\Delta\tilde{\rho}$ (non-smoothed) variation.

**Supplementary Note 3**

***Operando* Raman spectroscopy** was conducted using the WITec Alpha 300R (Germany) confocal Raman microscope with a laser wavelength of 532 nm. Laser energy was calibrated using a Si calibration sample. A compression-controlled Raman three-electrode battery cell (Redoxme AB, Sweden) with a sapphire window was used for *operando* measurements. The setup includes an electrode composed of an 8:1:1 ratio of active material, conductive carbon, and PVDF binder, cast on an aluminum current collector, along with a separator and a thin sodium foil counter/reference electrode with a small hole in the center, creating a two-electrode sandwich cell configuration. This approach eliminates the need for a free-standing electrode, as the electrode is directly targeted through the hole in the sodium. Spectra were collected from areas close to the sodium. A BioLogic SP-200 (France) portable potentiostat was utilized for electrochemical measurements. Sodiation and desodiation processes were conducted at 90 mA $g^{-1}$ current density. Spectra were collected every 15 minutes instead of continuous acquisition to avoid damaging the sample and electrolyte. A laser power of 1 mW was selected for the acquisitions with a 20-sec. integration time. Data refinement was conducted by subtracting the background and smoothing the signal (adjacent average) using WITec Project Five 5.2 software. The Raman signature of the as-prepared HC powder and electrode is shown in **Figures S11a** and **S11b**, respectively. A Raman spectrum of the electrode in the electrochemical Raman cell and the same spectrum after background subtraction are shown in **Figures S11c** and **S11d**, respectively. **Figure S12** presents the raw data during sodiation and desodiation, while **Figure S13** displays a heat map of the non-normalized data. The latter reveals a distinct decrease and increase in the intensity of the *D*-bands, along with a downshift in the *G*-band.

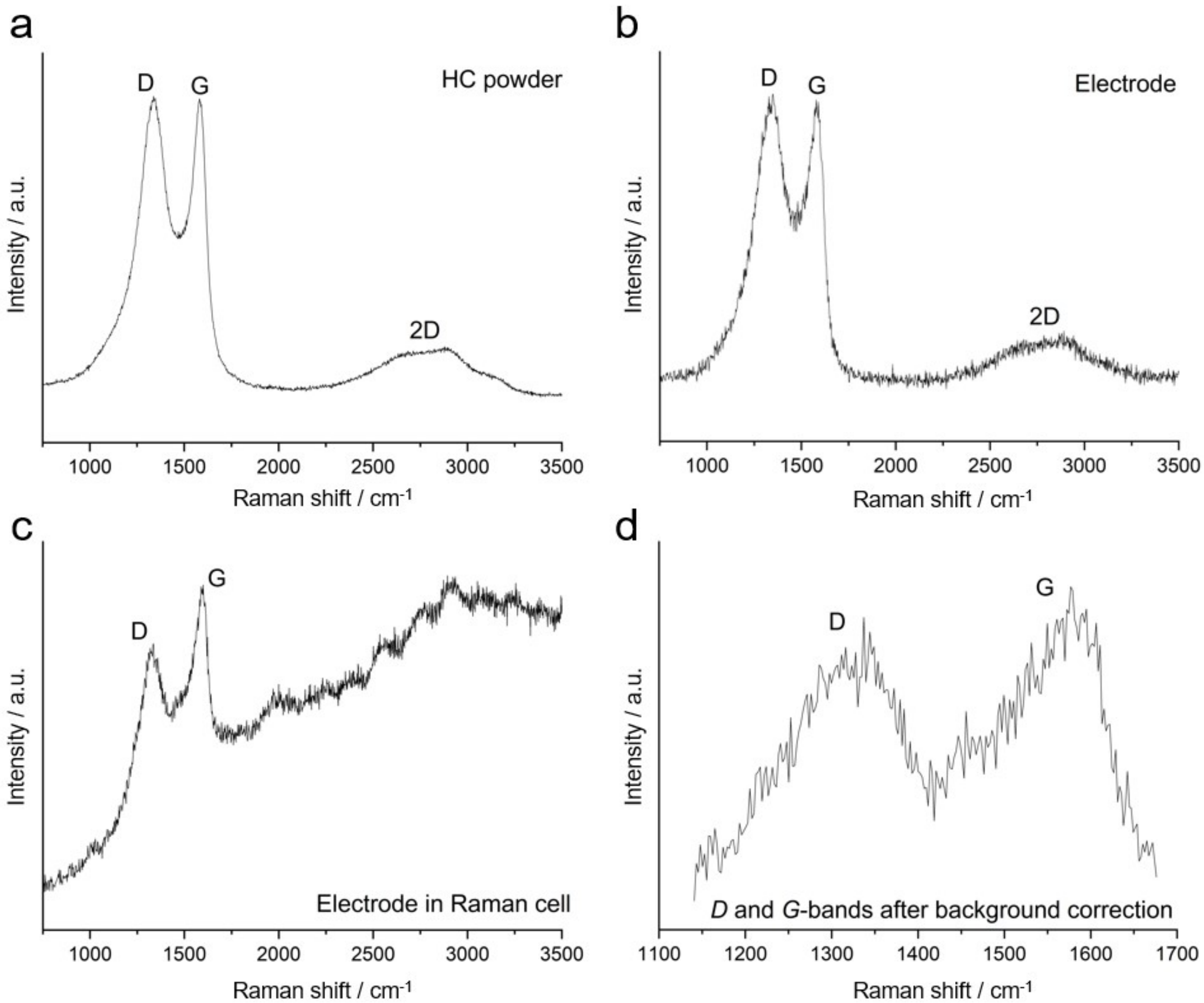


**Figure S11. (a)** Raman spectrum of the as-prepared HC powder. **(b)** Raman spectrum of the electrode. **(c)** Raman spectrum of the electrode in a Raman cell through the sapphire window and electrolyte, showing a fluorescence background. **(d)** *D*- and *G*-bands after background subtraction and data refinement.

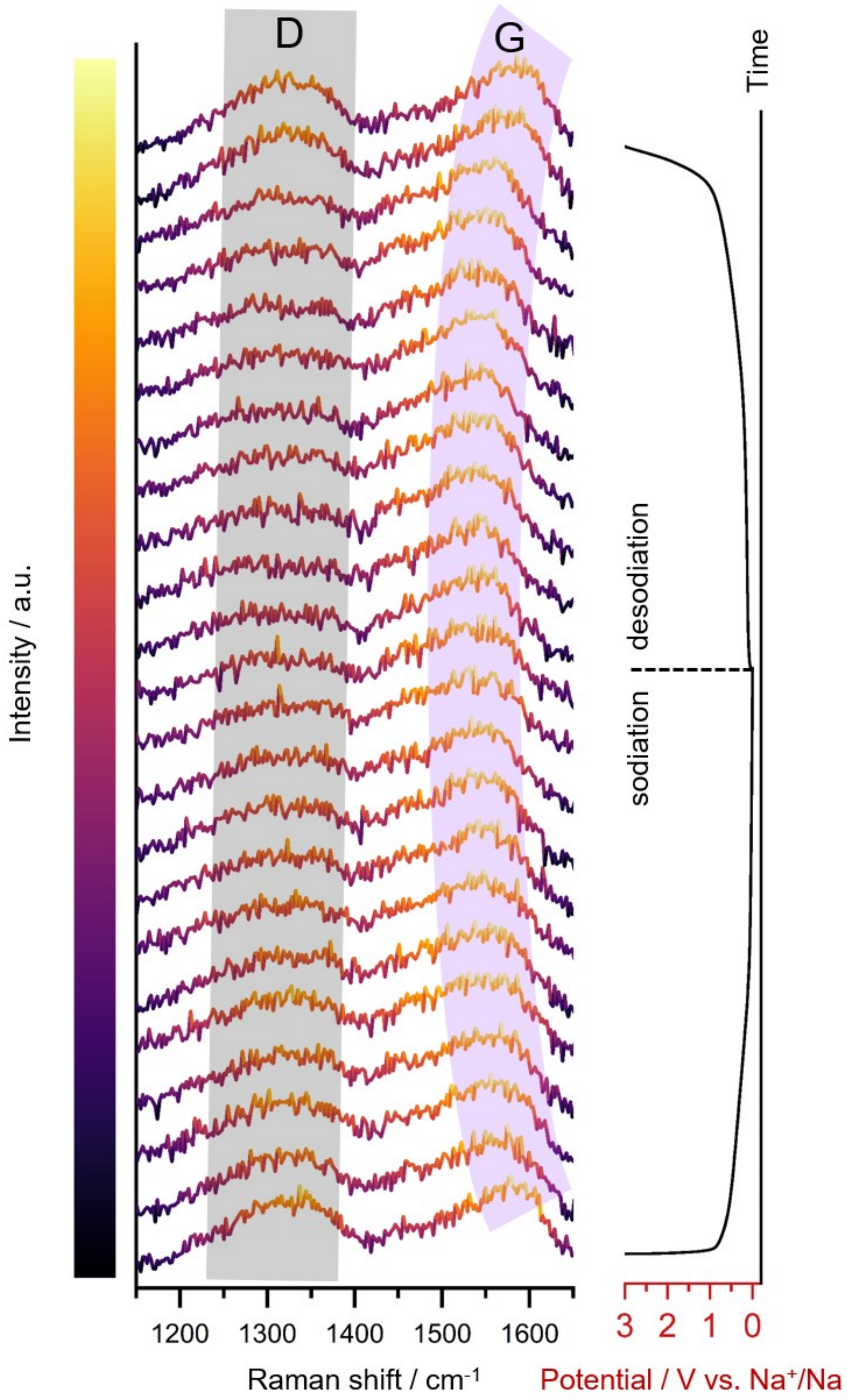


**Figure S12.** Non-smoothed Raman spectra of the HC during sodiation and desodiation, revealing slight intensity changes in the *D*-band and a sharp shift in the *G*-band.

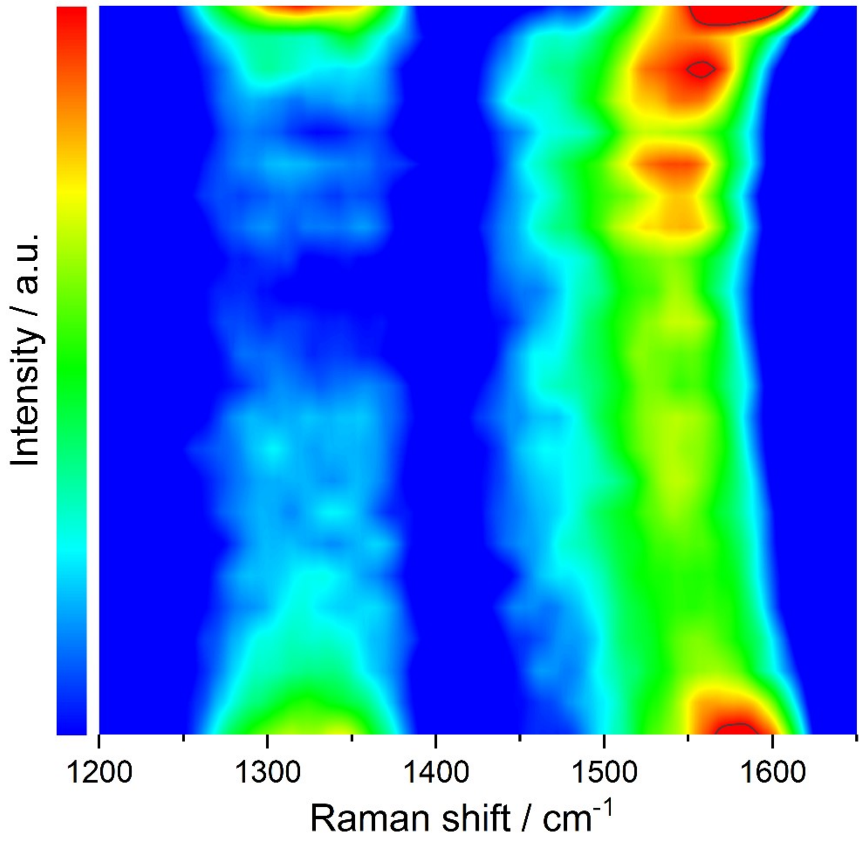


**Figure S13.** A non-normalized heat map visualizes the intensity change of the *D*-band and a pronounced shift in the *G*-band during sodiation and desodiation.

## Supplementary Note 4

***Operando* wide-angle X-ray scattering (WAXS)** measurements were conducted using a Bruker Nanostar II instrument (USA) with Cu Kα radiation (λ = 0.154 nm). The sample-to-detector distance was approximately 57 mm, covering a *q*-range of 3.5 to 29.5 $nm^{-1}$. Calibration of the scattering vector was performed using a glassy carbon standard (NIST SRM3600). The beam width was approximately 115 μm, allowing for localized structural probing across the electrode. Spectra were collected every 30 minutes during sodiation/desodiation at a current density of 30 mA $g^{-1}$ using a BioLogic SP-200 potentiostat.

The same custom cell configuration used for operando SAXS measurements was employed here (**Figure S2**), using binder- and additive-free electrodes. A key advantage of this approach is that the electrode does not need to be self-standing, as is often required in operando XRD (Bragg-Brentano geometry) studies. This eliminates background contributions from amorphous binders and conductive additives, which can overlap with signals from disordered carbons. To further reduce interference from non-active components, a 2 mm window was introduced at the center of the GF/C separator. A 2D transmission intensity map was used to locate this region, allowing precise alignment of the beam to collect WAXS signals predominantly from the HC electrode (**Figure S14a**).

To assess potential background overlap from the electrolyte, WAXS data were also collected for the liquid electrolyte sealed in a capillary. As shown in **Figure S14b**, the electrolyte exhibits a broad peak centered at ~14.8 $nm^{-1}$, well separated from the C(002) reflection of hard carbon (~18 $nm^{-1}$). Given the minimal volume of electrolyte present in the operando cell, the contribution of this signal is estimated to be negligible. Still, to quantitatively deconvolute any overlap, PseudoVoigt fitting was applied to *operando* WAXS spectra, isolating the contributions of the C(002) peak and electrolyte background (**Figure S14c**). This analysis confirms that the electrolyte does not interfere with the C(002) peak intensity or position.

Data reduction involved azimuthal integration of 2D patterns to obtain 1D scattering curves, followed by background subtraction, excluding the active material. For visualizing peak evolution, the C(002) intensity was normalized to the initial pattern at OCV. All scattering data were corrected for transmission and primary beam fluctuations. A second experimental run was conducted with another HC electrode under identical conditions to confirm reproducibility, which showed consistent results (**Figure S15**).

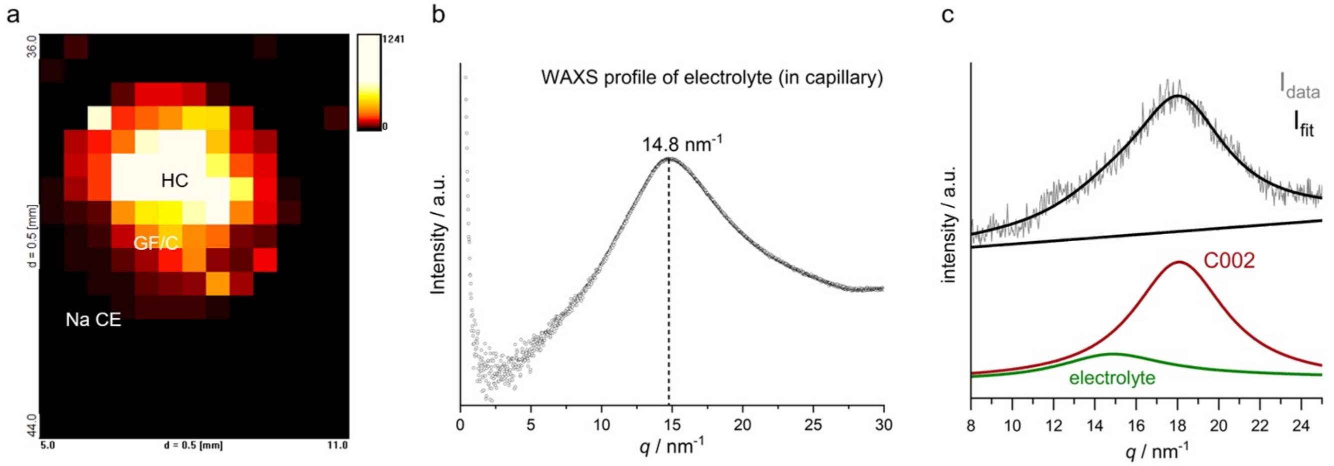


**Figure S14. (a)** Transmission intensity map of the operando WAXS cell showing the location of the HC electrode, GF/C separator, and sodium counter electrode. The ~2 mm hole in the separator allowed direct information from the HC electrode, minimizing background contributions. **(b)** WAXS profile of the electrolyte measured in a sealed capillary, displaying a broad scattering feature centered at ~14.8 $nm^{-1}$. **(c)** PseudoVoigt deconvolution of a representative operando WAXS spectrum. The C(002) peak is separated from electrolyte-induced scattering, confirming that the electrolyte background does not significantly affect the peak position.

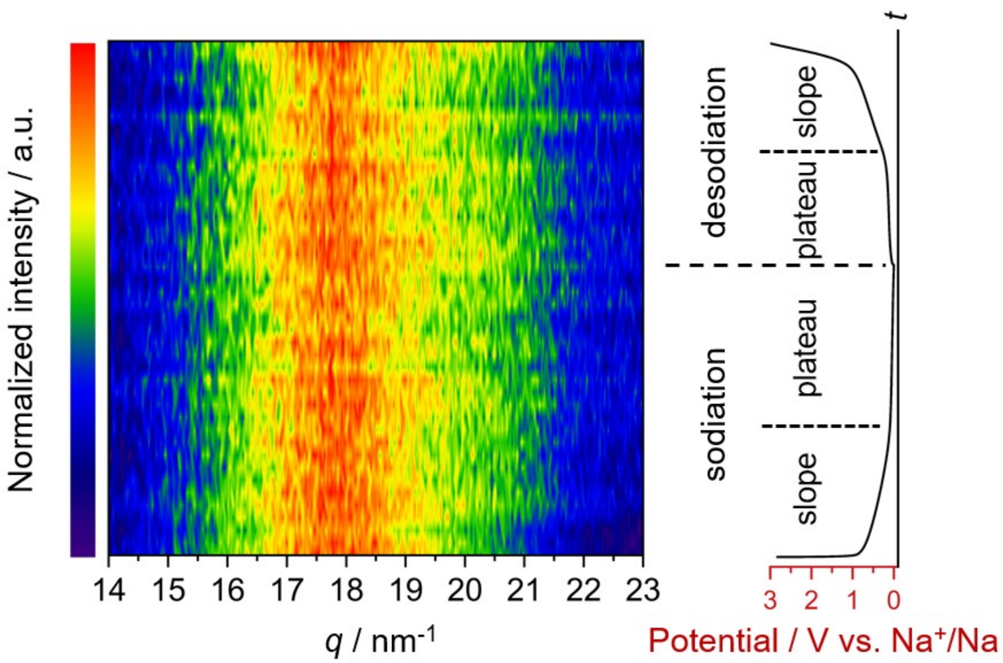


**Figure S15.** Heat map visualizing no pronounced shift in the C(002) peak also in the reproducibility experiment.

## Supplementary references

1. Eren EO, Esen C, Scoppola E, Song Z, Senokos E, Zschiesche H, et al. Microporous Sulfur–Carbon Materials with Extended Sodium Storage Window. Adv Sci. 2024;11(16):2310196.

2. Weppner W, Huggins RA. Determination of the Kinetic Parameters of Mixed-Conducting Electrodes and Application to the System Li3Sb. J Electrochem Soc. 1977;124(10):1569.

3. Delacourt C, Ati M, Tarascon JM. Measurement of Lithium Diffusion Coefficient in LiyFeSO4F. J Electrochem Soc. 2011;158(6):A741.

4. Horner JS, Whang G, Ashby DS, Kolesnichenko IV, Lambert TN, Dunn BS, et al. Electrochemical Modeling of GITT Measurements for Improved Solid-State Diffusion Coefficient Evaluation. ACS Appl Energy Mater. 2021;4(10):11460-9.

5. Jian Z, Xing Z, Bommier C, Li Z, Ji X. Hard Carbon Microspheres: Potassium-Ion Anode Versus Sodium-Ion Anode. Adv Energy Mater. 2016;6(3):1501874.

6. Wang K, Jin Y, Sun S, Huang Y, Peng J, Luo J, et al. Low-Cost and High-Performance Hard Carbon Anode Materials for Sodium-Ion Batteries. ACS Omega. 2017;2(4):1687-95.

7. Li Y, Hu Y-S, Titirici M-M, Chen L, Huang X. Hard Carbon Microtubes Made from Renewable Cotton as High-Performance Anode Material for Sodium-Ion Batteries. Adv Energy Mater. 2016;6(18):1600659.

8. Deiss E. Spurious chemical diffusion coefficients of Li+ in electrode materials evaluated with GITT. Electrochim Acta. 2005;50(14):2927-32.

9. Erko A, Zizak I. Hard X-ray micro-spectroscopy at Berliner Elektronenspeicherring für Synchrotronstrahlung II. Spectrochimica Acta Part B: Atomic Spectroscopy. 2009;64(9):833-48.

10. Kieffer J, Karkoulis D. PyFAI, a versatile library for azimuthal regrouping. Journal of Physics: Conference Series. 2013;425(20):202012.

11. Saurel D, Segalini J, Jauregui M, Pendashteh A, Daffos B, Simon P, Casas-Cabanas M. A SAXS outlook on disordered carbonaceous materials for electrochemical energy storage. Energy Storage Mater. 2019;21:162-73.
